# Supplementary material for: Heterochromatin Dynamics during the Differentiation Process Revealed by the DNA Methylation Reporter Mouse, MethylRO
Source: Stem Cell Reports. 2014 Jun 3;2(6):910–24. doi: 10.1016/j.stemcr.2014.05.008 (PMC4050349; doi:10.1016/j.stemcr.2014.05.008)
Supplement: Document S1. Supplemental Experimental Procedures, Figures S1–S3, and Table S1 [file mmc1.pdf]

Stem Cell Reports, Volume *2*

Supplemental Information

Heterochromatin Dynamics during the Differentiation Process Revealed by the DNA Methylation Reporter Mouse, Methyl*RO*

Jun Ueda, Kazumitsu Maehara, Daisuke Mashiko, Takako Ichinose, Tatsuma Yao, Mayuko Hori, Yuko Sato, Hiroshi Kimura, Yasuyuki Ohkawa, and Kazuo Yamagata

Supplemental Figure Legends

Figure S1, related to Figure 4. Generation of the ROSA26-H2B-mCherry reporter mouse. (A) Targeting of H2B-mCherry to the *ROSA26* locus. Top to bottom: pBigT, a plasmid containing an FRT-flanked cassette with a promoter-less Neo selectable marker and a tpA transcriptional stop sequence, into which the H2B-mCherry was cloned; pROSA26PA, containing genomic *ROSA26* sequences for homologous recombination, and a diphtheria toxin gene (PGK-DTA) for negative selection in ES cells; the wild-type *ROSA26* locus, with the location of the primers indicated; structure of the targeted locus; and structure of the targeted locus after Flipase (FLP)-mediated excision of the FRT-flanked (Neo, tpA) cassette. The FRT sites are indicated by solid arrowheads. (B) Schematic representation of H2B-mCherry. (C) Validation of genotypes of ES cells by PCR. The insertion of short and long arms was confirmed by PCR using the primers indicated in (A). (D) Genotyping of the ROSA26-H2B-mCherry mouse by PCR. WT, wild-type; Het, heterozygous knock-in; Homo, homozygous knock-in. (E) The H2B-mCherry protein was expressed only when the neomycin resistance gene cassette was removed by FLP. The presence of H2B-mCherry was confirmed by immunoblotting using an anti-RFP antibody in ES cells. An anti-OCT4 antibody was used as a loading control. (F) ROSA26-H2B-mCherry was expressed ubiquitously in adult mouse organs. The panels show the expression of ROSA26-H2B-mCherry and CAG-EGFP genes in various organs, including the brain, heart, lung, kidney, pancreas, testis, spleen, liver, thymus, and seminal vesicles. Both control and ROSA26-H2B-mCherry mice were positive for EGFP because these mice were generated using green ES cells (Fujihara et al., 2013). Although CAG is considered to be ubiquitous, there was no obvious EGFP signal in liver.

Figure S2, related to Figure 5. (A) Three-dimensional images of a blastocyst-stage Methyl*RO* embryo. The arrow indicates a cell with no obvious mCherry-MBD-NLS foci. A confocal microscope equipped with a 60× silicone oil-immersion objective lens was used to obtain the z-axis images, and three-dimensional images were generated computationally using the MetaMorph software. Numbers indicate angles. (B) Time-lapse imaging of Methyl*RO* ES cells. Whole chromosomes were labeled with H2B-EGFP expressed from the *ROSA26* locus. Note that the mCherry-MBD-NLS probe was highly enriched in pericentromeric regions (arrows). Furthermore, mCherry-MBD-NLS foci remained visible throughout the cell cycle. This was in sharp contrast with the preimplantation embryonic development described in Figure 4, in which mCherry-MBD-NLS foci disappeared in G2 phase of cell cycle. ES cells were cultured in LIF and FCS-containing medium, and photos were taken every 10 min using a confocal microscope equipped with a 60× silicone oil-immersion objective lens as described in Supplemental Experimental Procedures. The time after starting observation is indicated at the bottom right of the panel (hour:minute).

Figure S3, related to Figure 6. Generation of *Oct4-EGFP* knock-in mice. (A) Targeting of OCT4-EGFP to the *Oct4* gene locus. Targeting vector was kindly gift of Dr. Hitoshi Niwa (RIKEN CDB) (Toyooka et al., 2008). (B) Validation of genotypes by PCR in ES cells. The insertion of short and long arms was confirmed by PCR using the primers indicated in (A). Knock-in mouse was generated using these ES cells. (C) Genotyping of *Oct4-EGFP* knock-in mice by PCR. (D) OCT4-EGFP protein expression was confirmed by western blotting using ES cell whole protein extracts. WT, wild-type; Het, heterozygous knock-in. (E) Time-lapse images of an *Oct4-EGFP* knock-in embryo (upper embryo). Bottom embryo is negative for *Oct4-EGFP*. The time after starting observation is indicated at the bottom of the panel (hour). OCT4-EGFP signal becomes visible from four-cell stage embryo (Supplemental Movie 6). (F) Confocal image of *Oct4-EGFP* knock-in ES cells. (G) Confocal images of H2B-EGFP- and mCherry-MBD-NLS-expressing ES cells and differentiated cells. Differentiation was induced by culturing ES cells with retinoic acid containing medium for 7 days. (H) Heterochromatin index can be induced using H2B-EGFP probe. Heterochromatin indexes of ES and differentiated cells were calculated using either mCherry-MBD-NLS or H2B-EGFP probes. Statistical significance was calculated using Mann-Whitney U test. (I) Normalization of mCherry-MBD-NLS with H2B-EGFP signals cancel out the difference between ES and differentiated cells. Statistical significance was calculated using Mann-Whitney U test.

Supplemental Table

Supplemental Table S1. Genotyping primers used in this study.

	Primer Name	Sequence
1	ROSA26-Short Arm-Fw	5′-CCT AAA GAA GAG GCT GTG CTT TGG-3′
2	MBD-Rev	5′-TTA CGG GGC CTC CTT CCT GAC CTC ACC ACT CT-3′
3	MBD-Fw	5′-AAG ACT CGG AAA CGT CAG GTT GGA CCC CAG-3′
4	ROSA26-Long Arm-Rev	5′-GTA GTT ACT CCA CTT TCA AGT TCC TTA TAA-3′
5	ROSA26-NotI-Fw	5′-GAG CGG CCG CCC ACC CTC CCC TTC CTC TGG-3′
6	ROSA26-NruI-Rev	5′-CCT CGC GAC ACT GTA TTT CAT ACT GTA GTA-3′
7	H2B-Rev	5′-TGC GCC TTA GTC ACC GCC TTC TTG GAG CCC-3′
8	mCherry-Fw	5′-TCC ACC GGC GGC ATG GAC GAG CTG TAC AAG-3′
9	Oct4-Short Arm-Fw	5′-GGG CGG GGC CAG AGG TCA AGG CTA GAG GGT-3′
10	EGFP-Rev	5′-TTA CTT GTA CAG CTC GTC CAT GCC G-3′
11	EGFP-Fw	5′-ATG GTG AGC AAG GGC GAG GAG CTG TT-3′
12	Oct4-Rev	5′-CCT CAG AGG CCA CTC AGC ACT CTC TCC TCT-3′
13	Oct4-Ex5-Fw	5′-GTG GTT CGA GTA TGG TTC TGT AAC CGG CGC-3′
14	Oct4-Ex5-Rev	5′-GCT ATC TAC TGT GTG TCC CAG TCT TTA TTT-3′

Supplemental Movies

Supplemental Movie S1, related to Figure 4. Time-lapse imaging of a Methyl*RO* embryo from the one-cell zygote to the expanded blastocyst stage. Zygotic activation of mCherry-MBD-NLS started from the two-cell stage.

Supplemental Movie S2, related to Figure 4. Three dimensional image of a blastocyst stage Methyl*RO* embryo.

Supplemental Movie S3, related to Figure 4. Time-lapse imaging of EGFP-CENP-C and mCherry-MBD-NLS in a Methyl*RO* embryo from the two-cell to the eight-cell stage.

Supplemental Movie S4, related to Figure 4. Time-lapse imaging of EGFP-PCNA and mCherry-MBD-NLS in a Methyl*RO* embryo from the two-cell to the eight-cell stage.

Supplemental Movie S5, related to Figure 4. Time-lapse imaging of H2B-EGFP- and mCherry-MBD-NLS-expressing ES cells.

Supplemental Movie S6, related to Figure 5. Time-lapse imaging of an *Oct4-EGFP* knock-in embryo from the one-cell zygote to the expanded blastocyst stage.

Supplemental Movie S7, related to Figure 5. Time-lapse imaging of the ES cell derivation process in an OCT4-EGFP and mCherry-MBD-NLS double-positive embryo.

Supplemental Movie S8, related to Figure 5. Time-lapse imaging of a trophectodermal cell differentiating into a trophoblast giant cell.

Supplemental Experimental Procedures

Cell culture

Mouse ES cells were grown on top of mouse embryonic fibroblasts and maintained in medium containing 20% fetal calf serum and 500 U/mL LIF. Cell culture was performed in 5% CO_2 at 37°C. ES cell derivation was performed as described previously (Yamagata et al., 2010). For differentiation of ES cells, cells were cultured in 10 μM all-trans retinoic acid (Nacalai Tesque Co., Ltd., Japan) and 10% fetal calf serum containing medium for 7 days.

MeDIP analysis

After fixation with 1% paraformaldehyde, cells were harvested with a cell scraper and samples were processed for MeDIP analysis. Briefly, the cross-linked chromatin was sheared by sonication (Bioruptor, COSMO BIO), and MNase treatment (final concentration 10 U/mL). The insoluble materials were removed by centrifugation at 15,000 rpm for 10 min and supernatants were diluted with ChIP buffer (10 mM Tris-HCl pH 8.0, 200 mM KCl, 1 mM $CaCl_2$, 0.5% NP40) containing protease inhibitors. Immunoprecipitations were performed overnight in 4°C using antibody-conjugated Dynabeads® (Life Technologies Inc., CA, USA), and mixtures were extensively washed with Wash buffer (10 mM Tris-HCl pH 8.0, 500 mM KCl, 1 mM $CaCl_2$, 0.5% NP40). The bound chromatin fragments were released from the beads by adding ChIP Elution buffer (50 mM Tris-HCl pH8.0, 10 mM EDTA, 1% SDS) and incubated in 50°C for 1 hour in the presence of 2% proteinase K. MeDIPed DNA was purified by PCR purification kit (Qiagen N.V., Netherlands) and sequenced by the HiSeq 1000 (Illumina, Inc., CA, USA). The reads (Input 57,423,576 reads; 5mC 58,942,039 reads; RFP 61,096,178 reads) were aligned to the mouse genome (mm9) using the bowtie (version 0.12.7) software (parameter: -v 3 -m 1) and only uniquely mapped reads were used for calculation (percentages of mapped reads were 75.5% for Input, 66.7% for 5mC, and 65.9% for RFP, respectively). The normalized ChIP-seq signal was calculated by subtracting the Reads Per Million mapped reads (RPM) (Mortazavi et al., 2008) of the Input signal from the IPed signal. The signals were calculated at 1 kbp genomic intervals with a 10 kbp window. Hilbert curves of the signals were generated using the HilbertVis software, as described previously (Anders, 2009). MeDIP-seq data were deposited with the GEO accession code GSE57024.

Generation of knock-in mice

For the generation of Methyl*RO* and *ROSA26-H2B-mCherry* mice, targeting vectors were linearized by *KpnI* and electroporated into C57BL/6N ES cells (Fujihara et al., 2013). Genotypes were verified by PCR and ES cells with normal karyotype were injected into eight-cell-stage Slc:ICR strain embryos to generate chimeric mice. Mature male chimeric mice were first crossed

with C57BL/6N female mice to obtain F1 mice; subsequently, the neomycin cassette was excised from the *ROSA26* locus with FLP to induce the expression of either mCherry-MBD-NLS or H2B-mCherry in the whole body. The following primers were used to genotype Methyl*RO* ES cells: ‘ROSA26-Short Arm-Fw’ and ‘MBD-Rev’ were used to detect the insertion of the short arm, and ‘MBD-Fw’ and ‘ROSA26-Long Arm-Rev’ were used to detect the insertion of the long arm. The following three primers were used to genotype the Methyl*RO* mouse: ‘ROSA26-NotI-Fw’, ‘ROSA26-NruI-Rev’, and ‘MBD-Rev’. To genotype *ROSA26-H2B-mCherry* ES cells, the following primers were used: ‘ROSA26-Short Arm-Fw’ and ‘H2B-Rev’ were used to detect the insertion of the short arm, and ‘mCherry-Fw’ and ‘ROSA26-Long Arm-Rev’ were used to detect the insertion of the long arm. The following three primers were used to genotype the *ROSA26-H2B-mCherry* mouse: ‘ROSA26-NotI-Fw’, ‘ROSA26-NruI-Rev’, and ‘H2B-Rev’. For the generation of the *Oct4-EGFP* knock-in mouse, a targeting vector described previously was used (Toyooka et al., 2008). The following primers were used to genotype *Oct4-EGFP* ES cells: ‘Oct4-Short Arm-Fw’ and ‘EGFP-Rev’ were used to detect the insertion of the short arm, and ‘EGFP-Fw’ and ‘Oct4-Rev’ were used to detect the insertion of the long arm. The following three primers were used to genotype the *Oct4-EGFP* knock-in mouse: ‘Oct4-Ex5-Fw’, ‘Oct4-Ex5-Rev’, and ‘EGFP-Rev’. Primer sequences are available in Supplemental Table S1. Methyl*RO*, conditional Methyl*RO*, *ROSA26-H2B-mCherry*, conditional *ROSA26-H2B-mCherry*, and *Oct4-EGFP* knock-in mice are available from the RIKEN BioResource Center. All animal experiments were approved by the Animal Care and Use Committee of the Research Institute for Microbial Diseases, Osaka University, Japan.

Antibodies

Anti-RFP (Cat. No. 632496, Takara Bio Inc., Japan), anti-GFP (Hayashi and Shirao, 1999), and anti-OCT4 (sc-5279, Santa Cruz Biotechnology, Inc., CA, USA) antibodies were used for western blotting. Anti-RFP (M165-3, MBL Co., Ltd., Japan) and anti-5mC (MAb-006-100, Diagenode Inc., USA) antibodies were used for MeDIP analysis.

Histology

E12.5 fetuses were obtained by crossing C57BL/6N females with Methyl*RO* heterozygous males and fixed with 4% paraformaldehyde at 4°C overnight. To prepare sections, fixed fetuses or organs were equilibrated in PBS (−) with 6.8% sucrose, dehydrated in acetone for 1 h, and embedded in glycol methacrylate (Technovit 8100; Heraeus Kulzer GmbH, Germany). Sections were cut at a thickness of 5 μm. For staining of testis section with recombinant EGFP-MBD-NLS probe, freshly prepared unfixed testes were embedded into O.C.T. compound (Sakura FineTek, Tokyo, Japan) and frozen. Then these samples were sectioned at a thickness of 8 μm, fixed with ethanol, blocked and stained with EGFP-MBD-NLS probe in 4.2 ng/mL concentration. All sections were stained with

Hoechst 33342 (Life Technologies Inc., CA, USA) and were imaged using an ECLIPSE Ti inverted confocal microscope (Nikon Co., Japan).

Generation of recombinant EGFP-MBD-NLS probe

EGFP-MBD-NLS was produced in bacteria and purified by column chromatography. EcoRI-NotI fragment containing EGFP-MBD from pcDNA3.1 GFP-MBD-NLS poly A (Yamagata et al., 2005) was cloned into pGEX-6P-1 (GE Healthcare) to express glutathione-S-transferase (GST) fusion protein. E. coli BL21 strain harboring the resulting plasmid was grown at 25°C and the expression of GST-EGFP-MBD-NLS was induced by addition of 0.1 mM isopropyl β-D-1-thiogalactopyranoside (7 h; 25°C). Cells were harvested by centrifugation, lysed in 50 mM Tris-HCl [pH 8.0], 150 mM NaCl, protease inhibitor cocktail (Nacalai Tesque), 0.2 mg/mL lysozyme (Seikagaku Kogyo), and 1% Triton X-100 by sonication. After centrifugation (8,900 ×g; 20 min; 4°C), the supernatant was mixed with Glutathione-Sepharose beads (GE Healthcare; 2 mL beads per 200 mL cell culture) overnight at 4°C. After washing beads with 0.1 M sodium phosphate [pH 7.0] buffer containing 1 M NaCl, GST-EGFP-MBD was eluted with 100 mM glutathione and digested with Prescision protease (31 units/mL; GE Healthcare) overnight at 4°C. GST moiety was absorbed by incubating with Glutathione-Sepharose beads. EGFP-MBD was further purified using a HiLoad Superdex75pg gel filtration column, concentrated up to 0.6 mg/mL in PBS, and stored at -80°C in small aliquots.

Live-cell imaging

Mouse oocyte preparation, *in vitro* fertilization (IVF), microinjection, and imaging were described previously (Yamagata et al., 2009; Yamagata and Ueda, 2013). Briefly, fertilized oocytes at the anaphase II-telophase II stage were injected with 100 μg/mL of EGFP-CENP-C (Kitajima et al., 2011) or 10 μg/mL of EGFP-PCNA (Leonhardt et al., 2000) probe mRNA, placed on the imaging system, and imaged at 37°C with 5% CO_2 in air conditions. A conventional inverted microscope (IX-71, Olympus Co., Japan) equipped with a Nipkow disk confocal unit (CSU-X1, Yokogawa Electric Co., Japan), electron-multiplying charge-coupled device camera (iXON3 DU897E-CS0-#BV-Y, Andor Technology plc., UK), silicon oil-immersion objective (30× or 60×) lenses (Olympus Co., Japan) was used for live-cell imaging. Live-cell imaging of the ES cell derivation process was performed as described previously (Yamagata et al., 2010).

Supplemental References

Anders, S. (2009). Visualization of genomic data with the Hilbert curve. Bioinformatics *25*, 1231-1235.

Fujihara, Y., Kaseda, K., Inoue, N., Ikawa, M., and Okabe, M. (2013). Production of mouse pups from germline transmission-failed knockout chimeras. Transgenic Res *22*, 195-200.

Hayashi, K., and Shirao, T. (1999). Change in the shape of dendritic spines caused by overexpression of drebrin in cultured cortical neurons. J Neurosci *19*, 3918-3925.

Kitajima, T.S., Ohsugi, M., and Ellenberg, J. (2011). Complete kinetochore tracking reveals error-prone homologous chromosome biorientation in mammalian oocytes. Cell *146*, 568-581.

Leonhardt, H., Rahn, H.P., Weinzierl, P., Sporbert, A., Cremer, T., Zink, D., and Cardoso, M.C. (2000). Dynamics of DNA replication factories in living cells. J Cell Biol *149*, 271-280.

Mortazavi, A., Williams, B.A., McCue, K., Schaeffer, L., and Wold, B. (2008). Mapping and quantifying mammalian transcriptomes by RNA-Seq. Nat Methods *5*, 621-628.

Toyooka, Y., Shimosato, D., Murakami, K., Takahashi, K., and Niwa, H. (2008). Identification and characterization of subpopulations in undifferentiated ES cell culture. Development *135*, 909-918.

Yamagata, K., Suetsugu, R., and Wakayama, T. (2009). Long-term, six-dimensional live-cell imaging for the mouse preimplantation embryo that does not affect full-term development. J Reprod Dev *55*, 343-350.

Yamagata, K., and Ueda, J. (2013). Long-term live-cell imaging of mammalian preimplantation development and derivation process of pluripotent stem cells from the embryos. Dev Growth Differ *55*, 378-389.

Yamagata, K., Ueda, J., Mizutani, E., Saitou, M., and Wakayama, T. (2010). Survival and death of epiblast cells during embryonic stem cell derivation revealed by long-term live-cell imaging with an Oct4 reporter system. Dev Biol *346*, 90-101.

Yamagata, K., Yamazaki, T., Yamashita, M., Hara, Y., Ogonuki, N., and Ogura, A. (2005). Noninvasive visualization of molecular events in the mammalian zygote. Genesis *43*, 71-79.

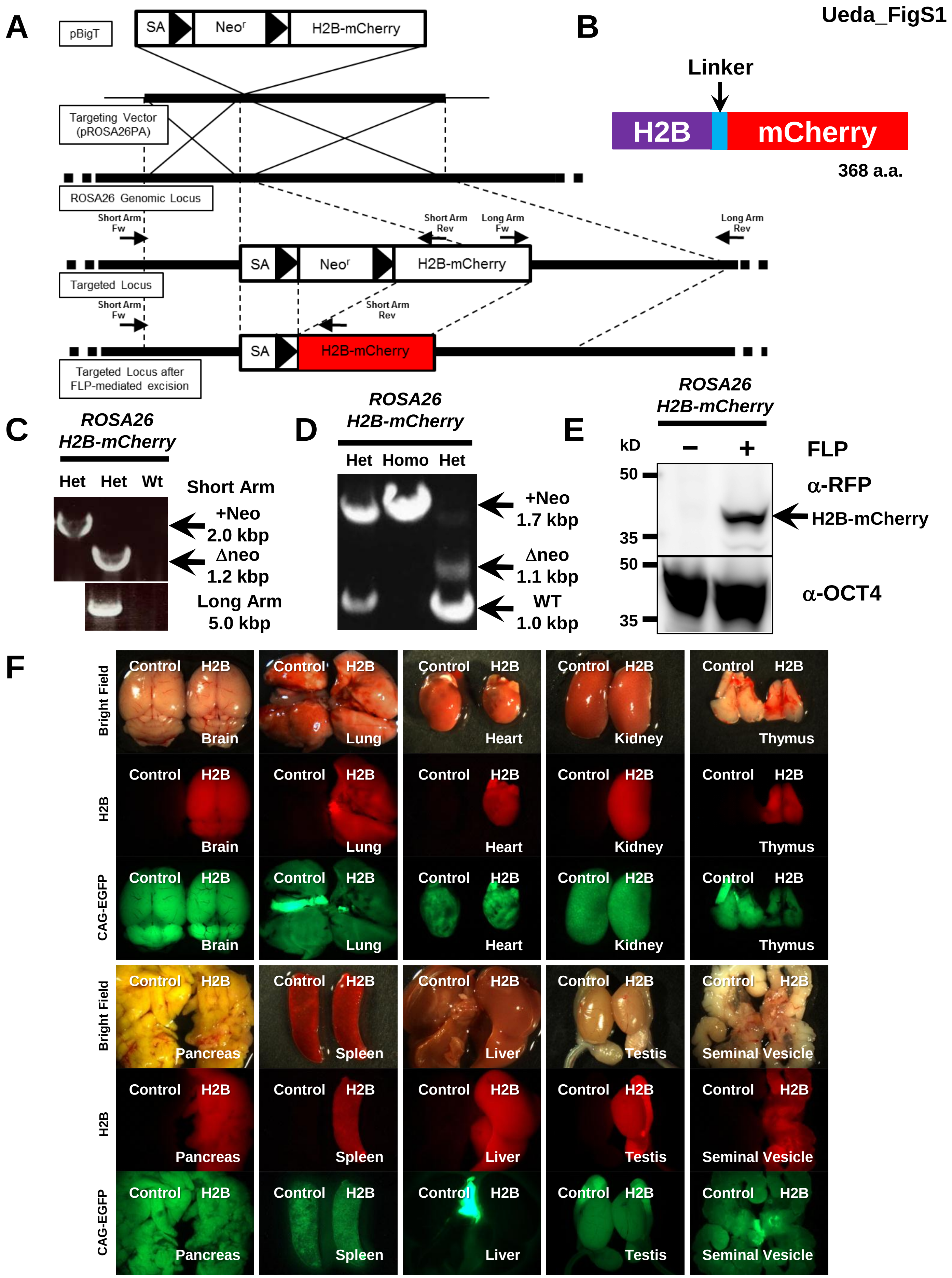

Ueda_FigS1
A
pBigT
SA
Neo^r
H2B-mCherry
Targeting Vector (pROSA26PA)
ROSA26 Genomic Locus
Short Arm Fw
Short Arm Rev
Long Arm Fw
Long Arm Rev
Targeted Locus
Targeted Locus after FLP-mediated excision
B
Linker
H2B
mCherry
368 a.a.
C
ROSA26 H2B-mCherry
Het Het Wt
Short Arm
+Neo 2.0 kbp
Δneo 1.2 kbp
Long Arm 5.0 kbp
D
ROSA26 H2B-mCherry
Het Homo Het
+Neo 1.7 kbp
Δneo 1.1 kbp
WT 1.0 kbp
E
ROSA26 H2B-mCherry
kD
− +
FLP
50
35
α-RFP
H2B-mCherry
α-OCT4
F
Bright Field
H2B
CAG-EGFP
Control
Brain
Lung
Heart
Kidney
Thymus
Pancreas
Spleen
Liver
Testis
Seminal Vesicle

Ueda_FigS2

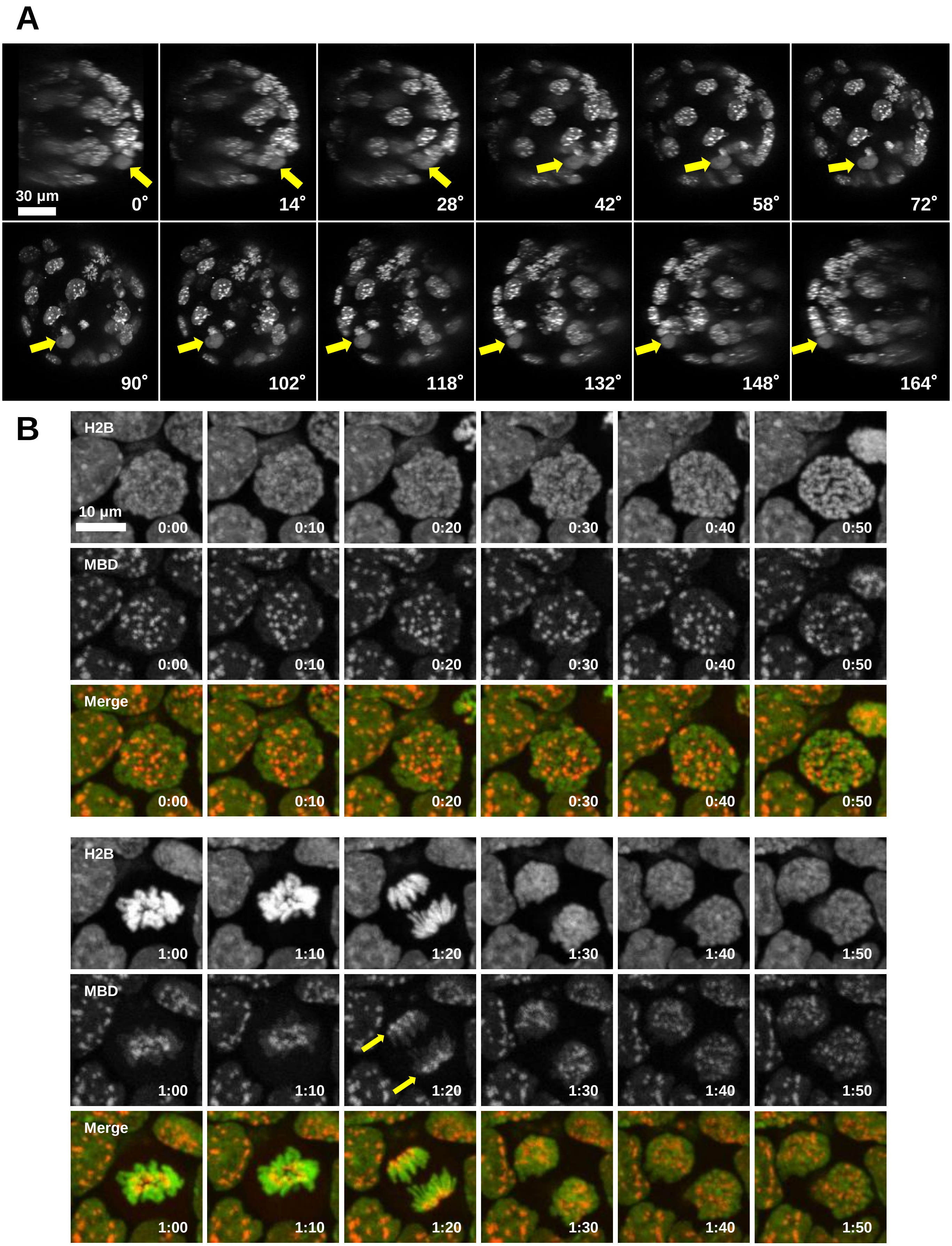
A
30 µm
0°
14°
28°
42°
58°
72°
90°
102°
118°
132°
148°
164°
B
H2B
MBD
Merge
10 µm
0:00
0:10
0:20
0:30
0:40
0:50
1:00
1:10
1:20
1:30
1:40
1:50

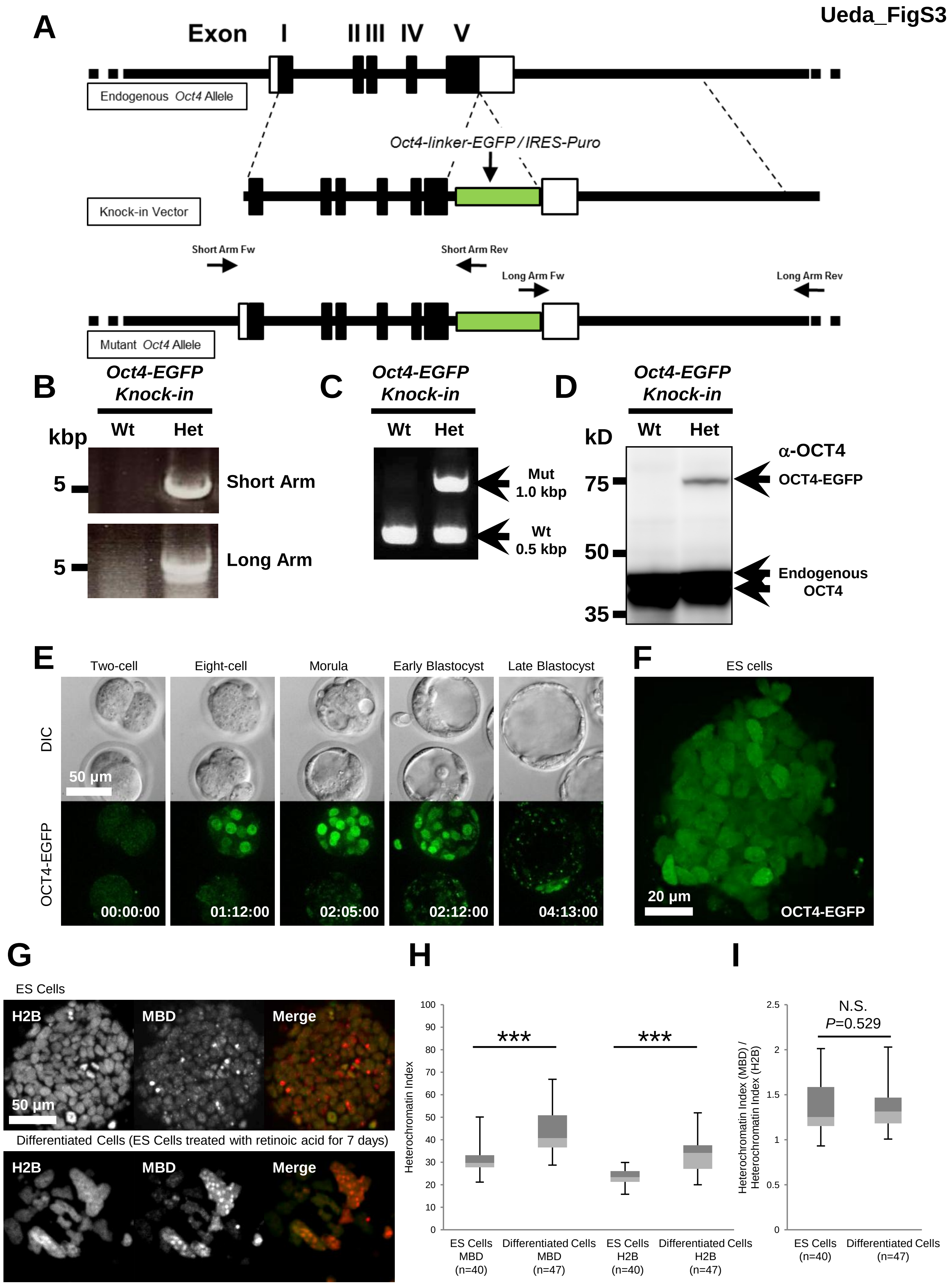
Ueda_FigS3
A
Exon I II III IV V
Endogenous Oct4 Allele
Oct4-linker-EGFP / IRES-Puro
Knock-in Vector
Short Arm Fw
Short Arm Rev
Long Arm Fw
Long Arm Rev
Mutant Oct4 Allele
B
Oct4-EGFP Knock-in
kbp
Wt
Het
5
Short Arm
5
Long Arm
C
Oct4-EGFP Knock-in
Wt
Het
Mut 1.0 kbp
Wt 0.5 kbp
D
Oct4-EGFP Knock-in
kD
Wt
Het
α-OCT4
OCT4-EGFP
75
50
35
Endogenous OCT4
E
Two-cell
Eight-cell
Morula
Early Blastocyst
Late Blastocyst
DIC
OCT4-EGFP
50 μm
00:00:00
01:12:00
02:05:00
02:12:00
04:13:00
F
ES cells
20 μm
OCT4-EGFP
G
ES Cells
H2B
MBD
Merge
50 μm
Differentiated Cells (ES Cells treated with retinoic acid for 7 days)
H2B
MBD
Merge
H
Heterochromatin Index
***
***
ES Cells MBD (n=40)
Differentiated Cells MBD (n=47)
ES Cells H2B (n=40)
Differentiated Cells H2B (n=47)
I
Heterochromatin Index (MBD) / Heterochromatin Index (H2B)
N.S.
P=0.529
ES Cells (n=40)
Differentiated Cells (n=47)
