# Supplementary material for: Heterochromatin Dynamics during the Differentiation Process Revealed by the DNA Methylation Reporter Mouse, MethylRO
Source: Stem Cell Reports. 2014 Jun 3;2(6):910–24. doi: 10.1016/j.stemcr.2014.05.008 (PMC4050349; doi:10.1016/j.stemcr.2014.05.008)
Supplement: Document S2. Article plus Supplemental Information [file mmc10.pdf]

Stem Cell Reports
Resource
OPEN ACCESS

# Heterochromatin Dynamics during the Differentiation Process Revealed by the DNA Methylation Reporter Mouse, Methyl*RO*

Jun Ueda,[1] Kazumitsu Maehara,[2] Daisuke Mashiko,[3] Takako Ichinose,[2] Tatsuma Yao,[4] Mayuko Hori,[1] Yuko Sato,[5] Hiroshi Kimura,[5] Yasuyuki Ohkawa,[2] and Kazuo Yamagata[1,*]
[1]Center for Genetic Analysis of Biological Responses, Research Institute for Microbial Diseases, Osaka University, 3-1 Yamadaoka, Suita 565-0871, Japan
[2]Department of Advanced Medical Initiatives, JST-CREST, Faculty of Medicine, Kyushu University, Fukuoka 812-8582, Japan
[3]Graduate School of Medicine, Osaka University, Suita 565-0871, Japan
[4]Research and Development Center, Fuso Pharmaceutical Industries, Ltd., Osaka 536-8523, Japan
[5]Graduate School of Frontier Biosciences, Osaka University, Suita 565-0871, Japan
*Correspondence: yamagata@biken.osaka-u.ac.jp
http://dx.doi.org/10.1016/j.stemcr.2014.05.008
This is an open access article under the CC BY license (http://creativecommons.org/licenses/by/3.0/).

## SUMMARY

In mammals, DNA is methylated at CpG sites, which play pivotal roles in gene silencing and chromatin organization. Furthermore, DNA methylation undergoes dynamic changes during development, differentiation, and in pathological processes. The conventional methods represent snapshots; therefore, the dynamics of this marker within living organisms remains unclear. To track this dynamics, we made a knockin mouse that expresses a red fluorescent protein (RFP)-fused *m*ethyl-CpG-*b*inding *d*omain (MBD) protein from the *ROSA26* locus ubiquitously; we named it Methyl*RO* (*methyl*ation probe in *ROSA26* locus). Using this mouse, we performed RFP-mediated methylated DNA immunoprecipitation sequencing (MeDIP-seq), whole-body section analysis, and live-cell imaging. We discovered that mobility and pattern of heterochromatin as well as DNA methylation signal intensity inside the nuclei can be markers for cellular differentiation status. Thus, the Methyl*RO* mouse represents a powerful bioresource and technique for DNA methylation dynamics studies in developmental biology, stem cell biology, as well as in disease states.

## INTRODUCTION

Methylation occurs at the cytosine base of CpG dinucleotides, to form 5-methylcytosine (5mC), which is sometimes called “the fifth nucleotide of DNA” based on its heritability. 5mC is frequently concentrated in repetitive sequences, such as pericentromeric regions and the transposable elements of normal somatic cells (Yamagata et al., 2007; Yoder et al., 1997), and is enriched in specific gene loci, such as differentially methylated regions of imprinted genes (Bird, 2002). Moreover, hypermethylation of CpG islands and hypomethylation of repeated DNA elements are key features of cancers (Bergman and Cedar, 2013; Ehrlich, 2002, 2009). Once these CpG sites are methylated, they are recognized by proteins of the *m*ethyl-CpG-*b*inding *d*omain (MBD) family, which then recruit additional protein complexes to these regions to repress gene expression and/or to generate a higher-order condensed chromatin structure called heterochromatin, to stabilize chromatin organizations (Bird, 2002; Clouaire and Stancheva, 2008). The importance of this epigenetic marker is highlighted by the fact that the loss of DNA methylation leads to early embryonic lethality in mice (around embryonic day 9.0 [E9.0]) (Li et al., 1992; Okano et al., 1999), and recent studies have indicated that DNA methylation undergoes dynamic changes during the mouse embryonic development, such as preimplantation and primordial germ cell development (Reik, 2007; Seki et al., 2005; Wu and Zhang, 2010), and stem cell differentiations (Yamaji et al., 2013). It is also well accepted that the DNA methylation status changes dramatically in tumorigenic and pathological processes in humans (Bergman and Cedar, 2013; Ehrlich, 2009), and it has become increasingly evident that the DNA methylation status changes dynamically in response to microenvironmental changes (Bergman and Cedar, 2013; Feil and Fraga, 2011; Waterland and Jirtle, 2004). For instance, the dietary intake in pregnant mice affects the DNA methylation status of transposable elements and imprinted genes in the offspring, but not in the mother, which then affects their susceptibility to diet-related chronic diseases (Waterland and Jirtle, 2003, 2004). Moreover, the discovery of proteins of the ten-eleven translocation (TET) family as 5mC hydroxylases further supports this notion that DNA methylation is dynamic (Guo et al., 2011; Ito et al., 2010; Wu and Zhang, 2010).

Although there is a strong need to investigate the dynamics of DNA methylation status, at present, there are no appropriate methods to study this epigenetic dynamics in living cells or, especially, in organisms. The major approaches available to date to study DNA methylation are bisulfite sequencing (Frommer et al., 1992), methylated DNA immunoprecipitation (MeDIP) combined either with microarray analyses or next-generation sequencing (MeDIP-seq) (Clark et al., 2012), and immunohistochemistry (Ito et al., 2010; Jørgensen et al., 2006). However, all of these techniques require fixation of the cells, hence

rendering it almost impossible to study the dynamics of this pivotal epigenetic marker, particularly in vivo. Furthermore, quantification using these methods usually requires at least a few thousand cells; thus, the results obtained are the average of the whole, and variations within specific populations are usually ignored in these experiments. In the case of immunohistochemistry, the intact chromatin structure is destroyed by the acid treatment that is required for the enhancement of the accessibility of the anti-5mC antibody to methylated DNA (Jørgensen et al., 2006).

Previously, we succeeded in visualizing the DNA methylation status by injecting a GFP-fused MBD of MBD1 protein (EGFP-MBD-NLS) mRNA into living mice zygotes. Using this method, we visualized the DNA methylation dynamics in preimplantation mouse embryos and found that DNA hypomethylation of major and minor satellites is a key signature that distinguishes germ cells from somatic cells (Yamagata et al., 2007; Yamazaki et al., 2007). Importantly, this method cannot only track epigenetic changes in four dimension, but can also achieve it at the single-cell resolution, which was difficult using the conventional methods. However, because mRNA injection was used to express the probe, these epigenetic live-cell imaging studies were limited to preimplantation-stage embryos (until morula stage), and advanced stages of embryonic and fetal development as well as organismal-level analyses were not possible.

Mouse embryonic stem cells (ESCs) were originally derived from the inner cell mass of the blastocyst (Evans and Kaufman, 1981), and these cells are developmentally advanced compared to early cleavage- and morula-stage embryos. This was indicated by the fact that trophoblast cells seldom arise from ESCs (Beddington and Robertson, 1989), even after they were cultured in chemical inhibitors "2i"-containing media to maintain naive pluripotency (Morgani et al., 2013). These observations implicate that irreversible epigenetic conversion has taken place in ESCs, whereas it was derived from early embryonic cells. Indeed, although recent studies using high-throughput DNA-sequencing analyses, either combined with bisulfite sequencing or chromatin immunoprecipitation (ChIP), have uncovered the epigenetic differences between preimplantation embryos and ESCs (Habibi et al., 2013; Smith et al., 2012; Yamaji et al., 2013), when these epigenetic changes occur and how these are reflected to 3D chromatin structures are still not known.

Here, to track the dynamic changes in this major repressive epigenetic marker during development and differentiation, especially in the ESC-derivation process, we have knocked in a red fluorescent protein (RFP)-fused MBD reporter probe (mCherry-MBD-NLS) into the *ROSA26* locus and generated a mouse strain that captures global DNA methylation status in living conditions. Using this reporter mouse, we have discovered that heterochromatin structure, which contains hypermethylated DNA, was highly dynamic in preimplantation embryo, whereas this dynamics was greatly reduced in pluripotent ESCs. We also found that this heterochromatin fixation occurred during the ESC-derivation process, revealed by live-cell imaging analyses. Thus, this model will become a powerful bioresource and technique for understanding DNA methylation dynamics in developmental biology, stem cell biology, and in disease states.

## RESULTS

### Generation of mCherry-MBD-NLS-Expressing ESCs

The cytomegalovirus (CMV) and "containing the chicken β-actin promoter and cytomegalovirus enhancer, β-actin intron and bovine globin poly-adenylation signal" (CAG) promoters are known for their strong and ubiquitous expression in mice. However, these promoters tend to yield heterogeneous gene expression, probably because of an uncertain position effect or random gene silencing of these transgenes within the mouse genome; in particular, they are not suitable for quantitative analysis in live-cell imaging (Figures 1A and 1C). For these reasons, we decided to knock in our gene of interest into a specific gene locus, to avoid heterogeneous gene expression. We chose the *ROSA26* locus because it is well known for its ubiquitous and uniform expression in mice (Soriano, 1999; Srinivas et al., 2001) and is widely used for reporter gene expression (Abe et al., 2011; Shioi et al., 2011). As shown in Figure 1D, we used the pBigT system to knock in mCherry-MBD-NLS (Srinivas et al., 2001). We fused RFP because it has a better signal-to-noise ratio and less self-fluorescence compared with GFP; in addition, it has low absorbance and light scattering and reaches tissues in depth, which render it more suitable for whole-body imaging (Figure 1B) (Shcherbo et al., 2007). Using a conventional gene-targeting method, the *mCherry-MBD-NLS* cDNA was successfully knocked into the *ROSA26* locus with high efficiency (28 out of 96). As expected, mCherry-MBD-NLS was only expressed when the neomycin-resistance gene cassette was excised by Flipase (FLP) (Figure 1F). In excellent agreement with a previous study by Kobayakawa et al. (2007), the mCherry-MBD-NLS probe localized to pericentromeric regions of chromosomes and formed foci in interphase nuclei in ESCs (Figure 1G). Importantly, the expression of the mCherry-MBD-NLS probe from *ROSA26* locus was uniform compared with probe expression from the CMV promoter (compare Figures 1G and 1C).

### MeDIP Using an Anti-RFP Antibody

Several lines of evidence have proven that the EGFP-MBD-NLS probe can recognize methylated DNA efficiently both

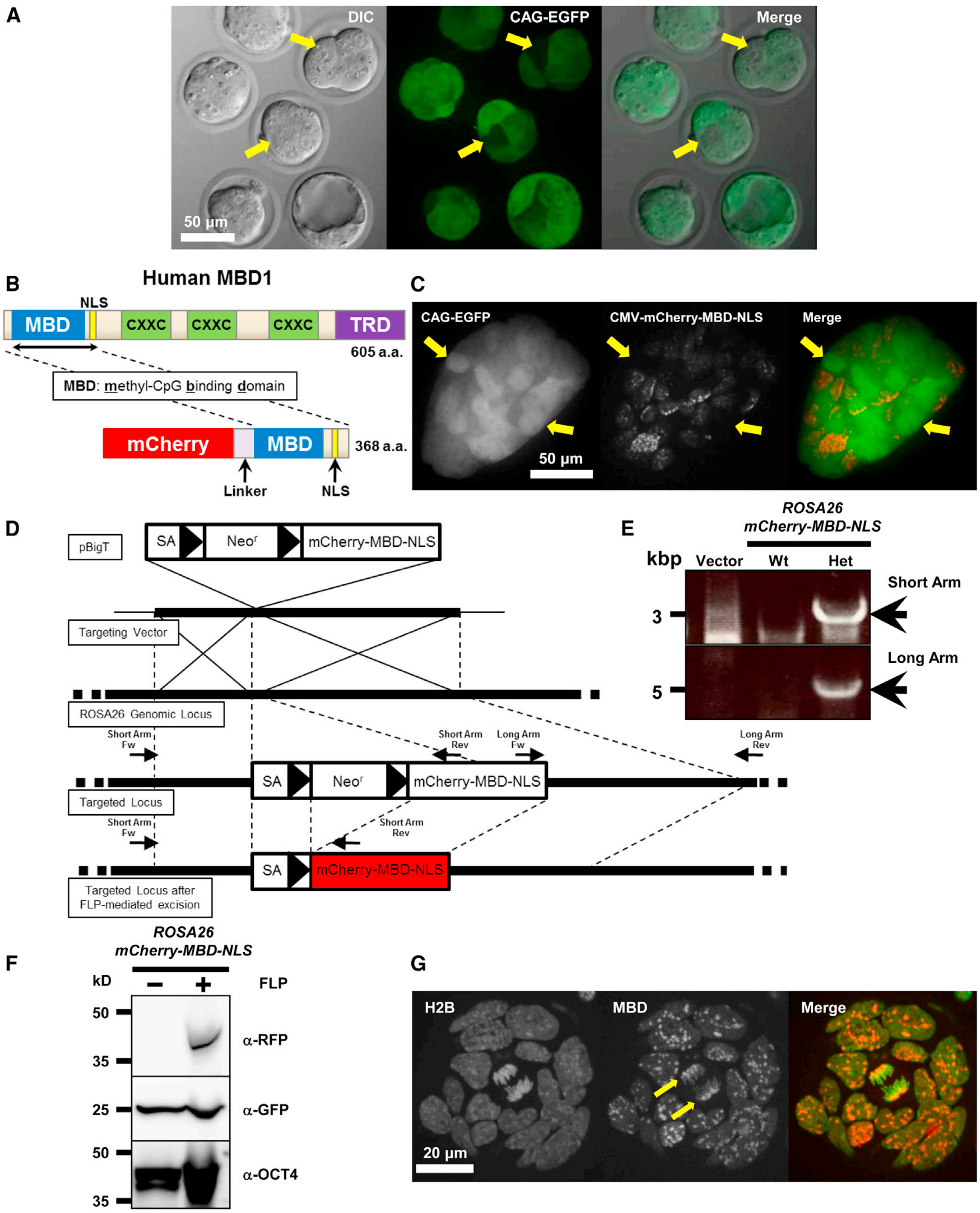


**Figure 1. Generation of mCherry-MBD-NLS-Expressing ESCs**

(A) CAG promoter yielded heterogeneous expression in preimplantation embryos. Morula-stage embryos from "Green mouse," which express EGFP from CAG promoter, are shown (Okabe et al., 1997). Note that some cells (arrows) had extremely low signals compared with surrounding cells. DIC, differential interference contrast.

(B) Schematic representation of hMBD1 and the mCherry-MBD-NLS protein structure. NLS, nuclear localization signal; CXXC, cysteine-rich domains; TRD, transcriptional repression domain.

(C) CMV promoter also yielded heterogeneous expression in ESCs that express EGFP from CAG promoter (Green ESCs). mCherry-MBD-NLS was expressed from the CMV promoter. Note that some cells (arrows) had no detectable RFP signals compared with surrounding cells.

*(legend continued on next page)*

in vitro and in vivo (Ohki et al., 2001; Tsumura et al., 2006; Yamazaki et al., 2007). For instance, Shirakawa and colleagues have resolved the structure of MBD binding to methylated DNA by nuclear magnetic resonance spectroscopy (Ohki et al., 2001), and we have previously demonstrated that MBD of human MBD1 (hMBD1) can bind specifically to methylated DNA by dot-blot analysis (Yamazaki et al., 2007). On the other hand, Okano and colleagues showed that EGFP-MBD-NLS probe does not form any foci within the nuclei of *Dnmt* triple-knockout ESCs (Tsumura et al., 2006). To further confirm the validity of this DNA methylation probe, we performed a MeDIP analysis in mCherry-MBD-NLS-expressing ESCs using a commercially available anti-RFP antibody. Intriguingly, anti-RFP antibody-mediated MeDIP yielded a highly specific enrichment of the methylated DNA fragments compared with conventional MeDIP analysis using an anti-5mC antibody (Figure 2A). We then processed these enriched DNA fragments for deep sequencing. As shown in Figures 2B and 2C, the overall RFP-mediated MeDIP-seq profile was very similar to that of the 5mC MeDIP-seq data and exhibited positive correlations on a genome-wide scale using different-sized windows (Pearson's correlation coefficients were 0.489 for 2 kbp, 0.597 for 5 kbp, and 0.690 for 10 kbp windows). The similarity was further visualized by HilbertViz software (Anders, 2009), which converts one-dimensional information into two dimensions, to present patterns visually (Figures 2D–2F). Importantly, the patterns of both RFP- and 5mC-mediated MeDIP-seq data were similar to that of the constitutive heterochromatin marker H3K9me3 ChIP sequencing (ChIP-seq) (Mikkelsen et al., 2007) and strikingly opposite to that of H3K9ac ChIP-seq, which marks active chromatin (Karmodiya et al., 2012) (Figures 2E and 2F). These data strongly support the hypothesis that the mCherry-MBD-NLS probe captures the heterochromatic regions within chromosomes. Next, we compared the enrichment of MeDIPed DNA on CpG islands. As shown in Figure 2G, the anti-RFP antibody precipitated a comparable amount of CpG island-containing DNA as that precipitated with the anti-5mC antibody (the Pearson's correlation coefficient of the reads per kilobase per million mapped reads [RPKMs] was 0.845), which indicated that RFP-mediated MeDIP-seq is comparable to 5mC-mediated MeDIP-seq. This result was further validated by comparing MeDIP-seq data with bisulfite-sequencing data of the CpG island from a previous report by Kobayashi et al. (2012). The CpG islands that were defined as heavily methylated by Kobayashi et al. (2012) (Figure 2G, the distribution indicated as red contour lines) located at RPKM with higher values, whereas CpG islands group with light levels of methylation (indicated as blue contour lines) located at lower end of intensity. Moreover, both 5mC-mediated MeDIP-seq and RFP-mediated MeDIP-seq were able to distinguish heavy- and light-level methylated CpG islands with high statistical significance (Student's t test p values were $1.50 \times 10^{-5}$ for 5mC and $1.95 \times 10^{-11}$ for RFP, respectively). Finally, MeDIP-seq data from 5mC and RFP were compared in closer detail. As shown in Figure 2H, the vast majority of signal patterns were strikingly similar between 5mC- and RFP-mediated MeDIP-seq data. Taken together, these data clearly demonstrated that the mCherry-MBD-NLS probe does indeed capture DNA methylation and that this epigenetic reporter can be used for RFP-mediated MeDIP analysis without using an anti-5mC antibody.

## MethylRO Mice Are Viable and Fertile

We then injected these ESCs into eight-cell-stage embryos of ICR mice to generate chimeric mice, and then male chimeric mice were crossed with C57BL/6 female mice to obtain *ROSA26-mCherry-MBD-NLS* heterozygous reporter mice. We further crossed these reporter mice with FLP-expressing transgenic mice to excise the neomycin cassette and induce *mCherry-MBD-NLS* expression (Figure 1D). As shown in Figures 3A and 3B, the mCherry-MBD-NLS-expressing mice were viable and fertile, indicating that the expression of this probe from the *ROSA26* locus has no obvious toxicity regarding mouse development, survival, and fertility. We named this epigenetic reporter mouse

(D) Targeting of mCherry-MBD-NLS to the *ROSA26* locus. From top to bottom are shown pBigT, a plasmid containing a FRT-flanked cassette with a promoter-less *Neo* selectable marker and a tpA transcriptional stop sequence, into which the *mCherry-MBD-NLS* was cloned; pROSA26PA, containing genomic *ROSA26* sequences for homologous recombination, and a diphtheria toxin gene (*PGK-DTA*), for negative selection in ESCs; wild-type *ROSA26* locus, with the location of the primers indicated; structure of the targeted locus; and structure of the targeted locus after FLP-mediated excision of the *FRT*-flanked (*Neo*, *tpA*) cassette. *FRT* sites are indicated by solid arrowheads.
(E) Validation of genotypes by PCR. The presence of short and long arms was confirmed by PCR using the primers indicated in (D). WT, wild-type; Het, heterozygous knockin. Primers used for genotyping are listed in Table S1.
(F) Confirmation of mCherry-MBD-NLS protein expression in mouse ESCs. ROSA26-mCherry-MBD-NLS ESCs were transfected transiently with the pCAGGS-FLP vector to excise the *Neo*$^r$ cassette (labeled as α-RFP). Because Green ESCs were used for knockin (Fujihara et al., 2013), these cells expressed EGFP (middle image). OCT4 was used as a loading control (bottom image).
(G) The mCherry-MBD-NLS probe localized to pericentromeric regions (arrows) and formed foci in interphase nuclei in ESCs. As an internal control, H2B-EGFP was knocked into to the other *ROSA26* locus.

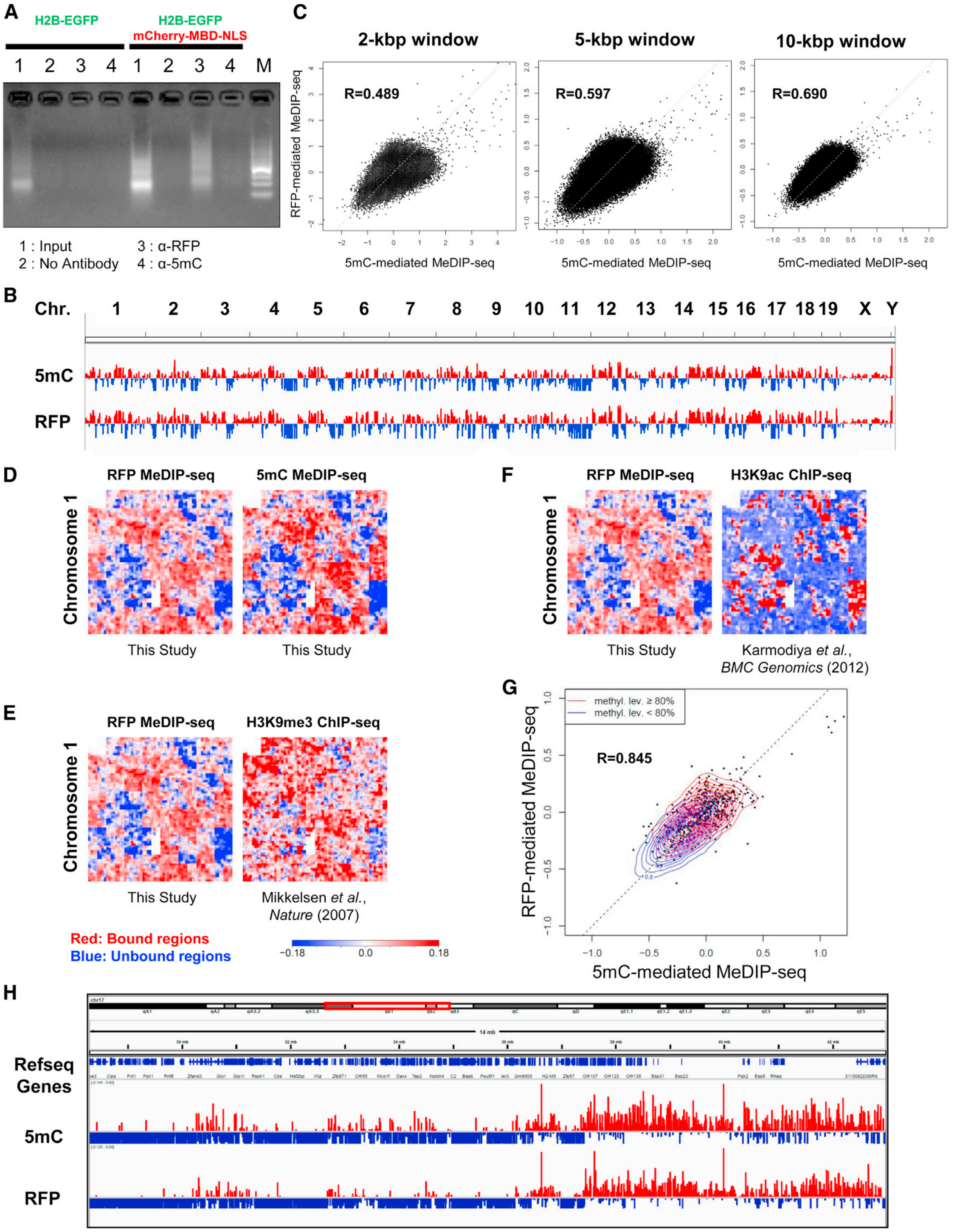


**Figure 2. Proper Binding of mCherry-MBD-NLS to Methylated CpG Revealed by RFP-Mediated MeDIP**

(A) MeDIP was performed either using anti-RFP or anti-5mC antibodies. ESCs expressing H2B-EGFP with and without mCherry-MBD-NLS were fixed and precipitated with the indicated antibodies, and precipitated DNA was run on a gel. M, 100 bp DNA ladder.

(B) RFP- and 5mC-mediated MeDIP-seq data of all chromosomes (Chr.) were compared using Integrative Genomics Viewer (Broad Institute, version 2.3.31). The MeDIPed signal was subtracted with Input signal, and positive and negative values are indicated by red and blue bars, respectively.

*(legend continued on next page)*

"MethylRO" as an abbreviation of "*methyl*ation probe in the *ROSA26* locus."

### Expression Profiles of the mCherry-MBD-NLS Probe in Fetal and Adult Mouse Organs and Tissues

Next, we examined various tissues and organs of adult and fetal Methyl*RO* mice. As expected, RFP signals were detected in all tissues examined, confirming that this probe is expressed ubiquitously within both fetal and adult mouse bodies (Figures 3C and 3D). Previously, the only way to detect DNA methylation in tissue sections was to immunostain the samples with an anti-5mC antibody. However, this procedure requires acid treatment (usually 4 N HCl for 15–20 min at room temperature) prior to antibody addition, and staining results vary depending on the duration of acid processing (Jørgensen et al., 2006). Therefore, to gain further insights on how this probe functions inside nuclei, we prepared tissue sections of an E12.5 fetus and observed it in closer detail (Figures 3E and 3F). Importantly, we observed a typical pattern of the mCherry-MBD-NLS probe inside nuclei in most somatic cells (compare Figures 1G and 3F), which implies that this probe is functional inside the mouse body, similar to that seen in ESCs. Furthermore, the mCherry-MBD-NLS probe colocalized with puncta in Hoechst-stained nuclei, which mark heterochromatic regions; this clearly indicated that the probe is functional and recognizes heterochromatin within the mouse body (Figure 3F).

We then went on to examine Methyl*RO* adult testicular sections because germ cells undergo dynamic epigenetic reprogramming during development and differentiation (Sasaki and Matsui, 2008; Seki et al., 2005; Yamagata et al., 2007). Surprisingly, a majority of the cells located inside seminiferous tubules exhibited weak fluorescent signals compared with surrounding somatic cells (myofibroblasts and Leydig cells) (Figure 4A). This was not due to the low or absent expression of the mCherry-MBD-NLS probe within germ cells because the fluorescence of H2B-mCherry expressed under the control of the *ROSA26* locus was observed in germ cells, from spermatogonia to elongating spermatids (Figure 4B; Figure S1 available online). Moreover, when we stained the testis section with recombinant EGFP-MBD-NLS protein, we found that this probe formed heterochromatic foci in somatic cells, but not in germ cells (Figure 4C). These results imply that the genomic DNA of germ cells is hypomethylated in pericentromeric regions, which supports our and other groups' previous findings (Filipponi et al., 2013; Yamagata et al., 2007). Taken together, these data clearly demonstrated that the Methyl*RO* mouse model can be used to study global DNA methylation patterns in tissues and organs of mice without using an anti-5mC antibody and acid treatment.

### Fluorescent Live-Cell Imaging of MethylRO Embryos

Finally, we performed live-cell imaging of preimplantation embryos collected from Methyl*RO* mice. As shown in Figure 5A and Movie S1, we have successfully imaged global DNA methylation dynamics throughout the preimplantation developmental stages. This was not possible when we injected EGFP-MBD-NLS probe mRNA into one-cell zygotes because the signal starts to decrease from the eight-cell stage onward, and it was extremely difficult or impossible to detect signals in blastocyst-stage embryos, probably because of the degradation of the probe, as observed in our previous reports (Yamagata et al., 2007; Yamazaki et al., 2007). The fluorescent signal started to become overt in the early two-cell stage and reached a maximum around the morula stage of embryos (Figures 5A and 6C), whereas heterochromatic foci were already visible from the two-cell stage. However, these foci were not as distinct as those of ESCs and somatic cells. Interestingly, the observation of blastocyst-stage Methyl*RO* embryos in closer detail revealed the presence of nuclei with no obvious foci (Figure S2A, arrow; Movie S2). This prompted us to look further into the dynamics of heterochromatin in preimplantation embryos.

(C) Comparison of RFP- and 5mC-mediated MeDIP-seq data by scatterplot diagram of normalized ChIP-seq signals. The axes of x and y are RPKM scores of 5mC and RFP MeDIP-seq, respectively. Each point indicates the scores at a same genomic region. The Pearson's correlation coefficients of the scores are 0.489 for a 2 kbp window, 0.597 for a 5 kbp window, and 0.690 for a 10 kbp window.

(D) MeDIP data were verified further using Hilbert curves. Methylated DNA regions are indicated in red, and negative values are indicated in blue, as in (B). Chromosome 1 is shown as an example.

(E) RFP-mediated MeDIP-seq data correlated positively with silent chromatin marker H3K9me3 ChIP-seq data. RFP-mediated MeDIP-seq data were compared with the results of a previous report (Mikkelsen et al., 2007) using Hilbert curves.

(F) RFP-mediated MeDIP-seq data correlated negatively with active chromatin marker H3K9ac ChIP-seq data. RFP-mediated MeDIP-seq data were compared with the results of a previous report (Karmodiya et al., 2012) using Hilbert curves.

(G) Enrichments of RFP- and 5mC-mediated MeDIP-seq signals were compared over CpG islands by scatterplot diagram. The axes of x and y are RPKM scores of 5mC- and RFP-mediated MeDIP-seq, respectively. Each distribution of heavily ($\geq$80%) and lightly (<80%) methylated CpG island groups, defined as previous report (Kobayashi et al., 2012), is indicated by red and blue contour lines, respectively. methyl. lev., methylated level.

(H) Closer comparison of 5mC- and RFP-mediated MeDIP-seq data. Genomic locus around *Pou5f1* gene is indicated.

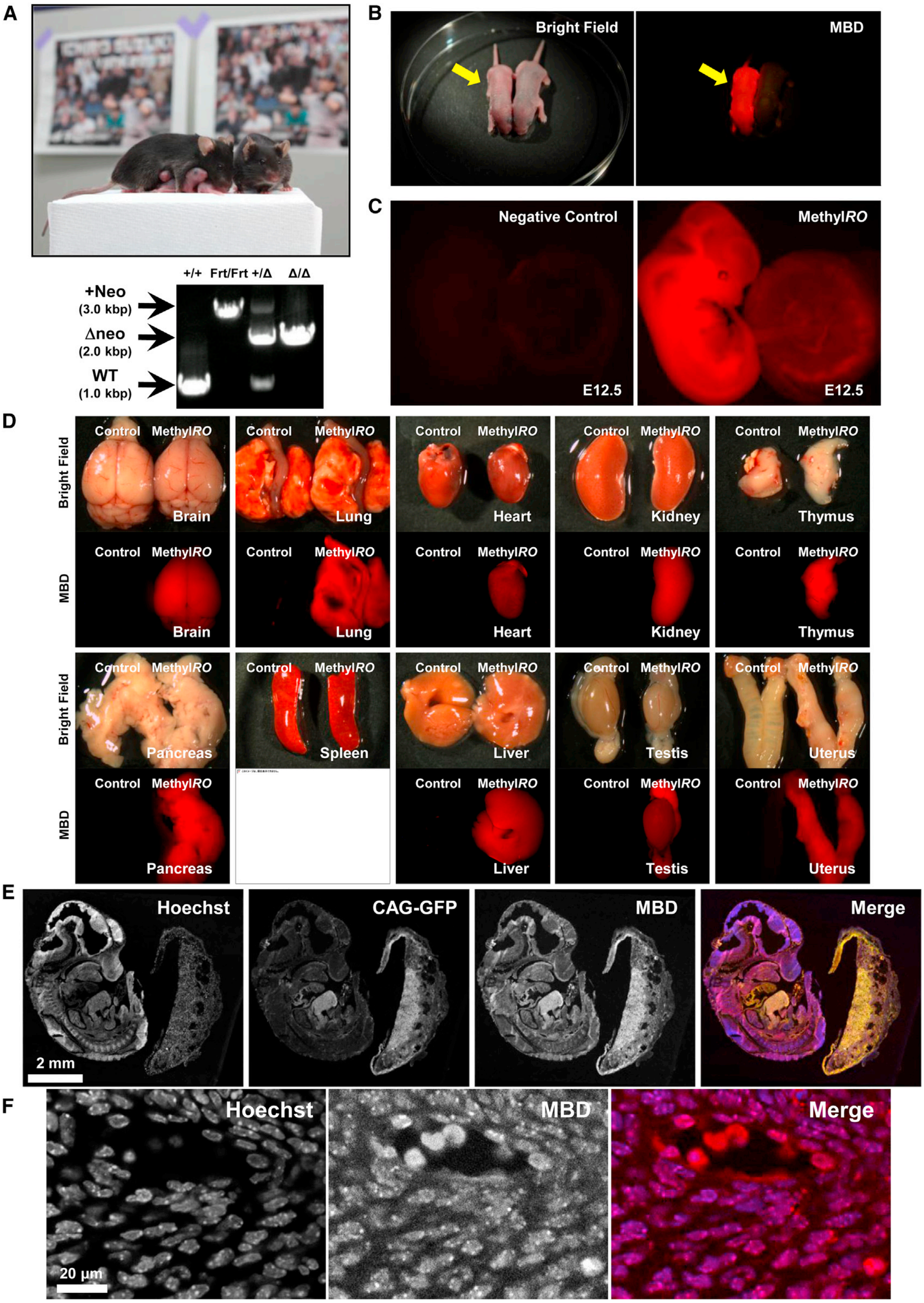


**Figure 3. Generation of Reporter Mouse Expressing mCherry-MBD-NLS in the Whole Body**

(A) Mice were viable, healthy, and fertile (top). Genotypes were verified by PCR using tail tip genomic DNA (bottom). +/+, wild-type; Frt/Frt, homozygous for FRT-floxed neomycin-resistant gene; +/Δ, heterozygous for mCherry-MBD-NLS; Δ/Δ, homozygous for mCherry-MBD-NLS. We named this reporter "Methyl*RO*," inspired by the famous Major League Baseball player Ichiro.

*(legend continued on next page)*

### Heterochromatin Is Highly Dynamic in Preimplantation Embryos

To analyze the nature of heterochromatin in preimplantation embryos, first, we imaged Methyl*RO* embryos together with the inner kinetochore protein CENP-C from the two-cell to eight-cell stages (Saitoh et al., 1992). In good agreement with the results of previous studies (Aguirre-Lavin et al., 2012; Yamazaki et al., 2007), the mCherry-MBD-NLS probe always localized adjacent to the CENP-C protein, indicating that the major foci that were visualized with this probe mark pericentromeric heterochromatin (Figures 5B and 5C, arrows) and form a chromocenter (Guenatri et al., 2004). However, surprisingly, we noticed that heterochromatin was highly dynamic and mobile in preimplantation embryos when it moved around inside interphase nuclei (Movie S3). This was also shown by projecting cellular nuclear images at time-axis direction; embryonic cells had vague and fewer heterochromatic foci compared to ESCs, indicating that these foci were more dynamic (Figure 5D). To quantify the heterochromatin movement, heterochromatic foci were tracked, and changes in distance between any two points were calculated in 5 min intervals up to 95 min, for a total of 19 time points. This method will allow us to rule out the movements and rotations of objects being measured (in our case, nuclei), which can affect the motion data (Miné-Hattab and Rothstein, 2012). As shown in Figure 5E, heterochromatic foci of embryonic cells (four-cell stage) showed more dynamic movement compared to that of ESCs and mouse embryonic fibroblast (MEF) cells. This result was further confirmed by calculating mobility of these foci, and embryonic heterochromatin showed statistically significant higher and varied velocity than those of ESCs and MEF cells (Figure 5F). To examine the relationship between heterochromatin dynamics and the cell cycle in preimplantation embryos, next, we imaged Methyl*RO* embryos together with the proliferating cell nuclear antigen (PCNA) probe (Leonhardt et al., 2000). As shown in Figure 5G, mCherry-MBD-NLS foci moved around dynamically during interphase (from the G1 to the S phases) and, importantly, colocalized with the PCNA at the late S phase. This result further confirmed that mCherry-MBD-NLS foci mark constitutive heterochromatin. However, unexpectedly, heterochromatic foci started to weaken from the G2 phase and had disappeared by the late G2 phase (Movie S4). This was in stark contrast with what was observed in ESCs because heterochromatic foci were observed in these cells throughout the cell cycle, and foci never disappeared (Figure S2B; Movie S5). Thus, the cell with no obvious foci shown in Figure S2A may be in the G2 phase of the cell cycle. These data strongly indicated that preimplantation embryonic heterochromatin is highly dynamic and that the nuclear organization and DNA methylation status of preimplantation embryos are different from those of ESCs, even though ESCs are considered to be close to the inner cell mass cells of blastocysts (Nichols and Smith, 2011).

### Heterochromatin Organization Changes Dynamically during the ESC-Derivation Process

The data described in the previous section imply that ESCs likely undergo epigenetic and heterochromatin remodeling during the derivation processes. To address this outstanding question, we have carried out a live-cell imaging analysis of the ESC-derivation process using Methyl*RO* embryos. According to a methodology described previously by us (Yamagata et al., 2010), morula-stage embryos collected from Methyl*RO* mice were placed on top of feeder cells, cultured in conventional ESC-derivation medium, and imaged every 30 min up to 7 days. To label the pluripotent epiblast cell lineage, we have generated *Oct4-EGFP* knockin mice using a targeting vector construct established previously by Toyooka et al. (2008) (Figures 6A and S3; Movie S6). Intriguingly, although heterochromatic foci were vague in the beginning, they became distinct during the course of derivation, which was confirmed by the quantification of the signal intensities and by calculating heterochromatin index, which is defined as a coefficient of variation of signal patterns (Figures 6A and 6D–6F; Movie S7). These data clearly indicated that epiblast cells have undergone epigenetic and heterochromatin remodeling during the ESC-derivation process. In parallel to the ESC-derivation process, we also captured the dynamic nuclear remodeling of trophoblast giant cells during their emergence (Figure 6B). Strikingly, the heterochromatic

(B) Neonate Methyl*RO* mice (left) are red; red fluorescence can be observed by the naked eye using a fluorescence filter (right, arrow).
(C) The mCherry-MBD-NLS probe was expressed ubiquitously in E12.5 fetuses. Negative control and Methyl*RO* fetuses were observed under a fluorescence stereoscopic microscope. Error bars indicate the SD.
(D) The mCherry-MBD-NLS probe was expressed ubiquitously in adult organs. mCherry-MBD-NLS expression was observed in the brain, heart, lung, kidney, pancreas, thymus, spleen, liver, testis, and uterus (labeled as Methyl*RO*). Error bars indicate the SD.
(E) Section of an E12.5 Methyl*RO* mouse fetus. Methyl*RO* E12.5 fetus expressing EGFP from CAG promoter was sectioned, stained with Hoechst 33342, and observed under a fluorescence confocal microscope (Nikon; ECLIPSE Ti inverted confocal microscope). The images taken were tiled together computationally using NIS-Elements viewer software (Nikon).
(F) Section of an E12.5 Methyl*RO* mouse fetus. Somatic cells near the tail were taken with a 40× objective lens. The mCherry-MBD-NLS probe within somatic cell nuclei exhibited a typical heterochromatic foci pattern.

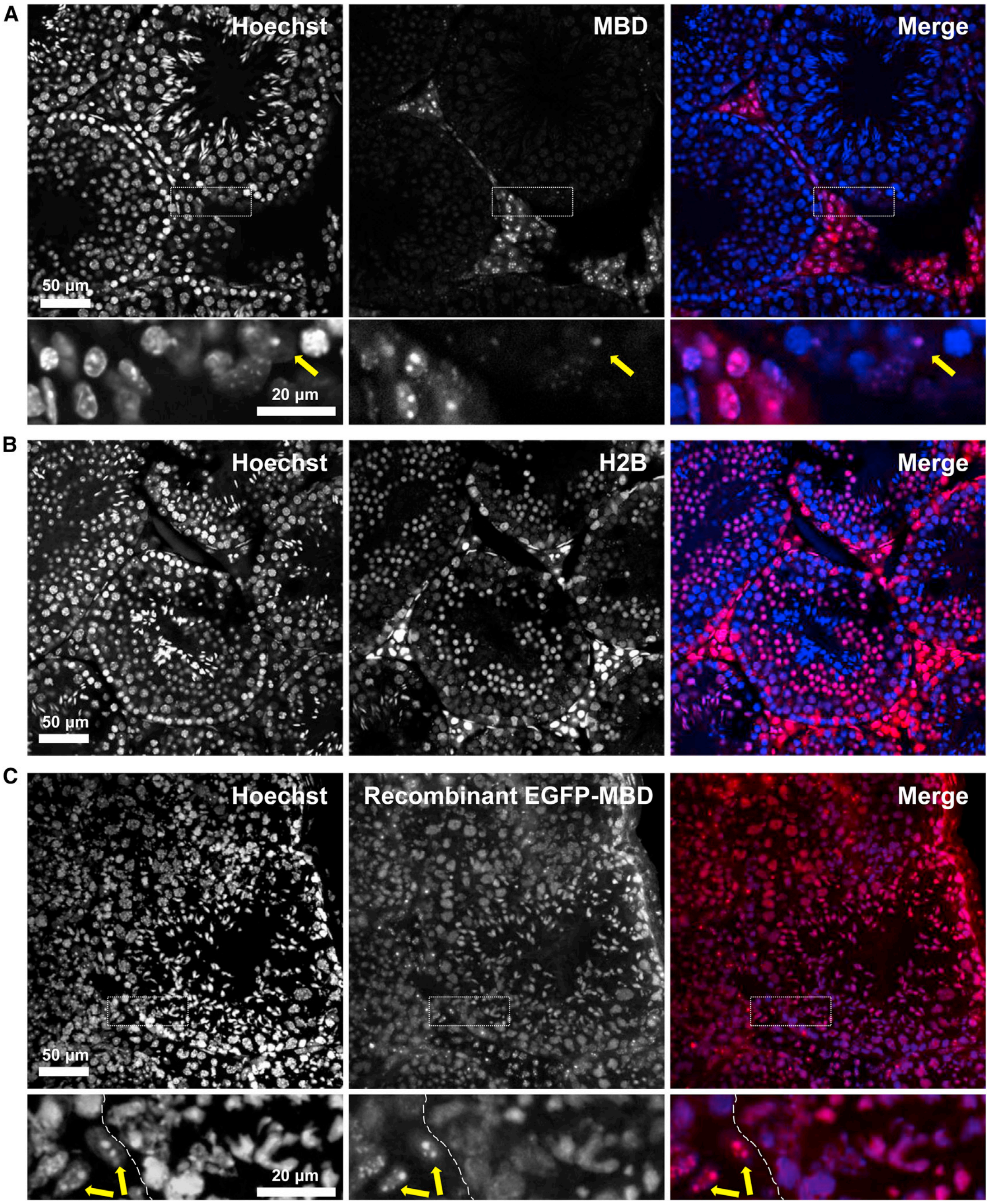


**Figure 4. Testicular Germ Cells Are DNA Hypomethylated in Adult Mice**

(A) Section of a Methyl*RO* mouse testis. Nuclei were stained with Hoechst 33342. Magnified images of boxed areas are shown on the bottom. Arrows indicate Sertoli cells.

(B) Section of a ROSA26-H2B-mCherry mouse testis. Nuclei were stained with Hoechst 33342. Note that H2B-mCherry signal can be detected from spermatogonia to elongating spermatids.

(C) Wild-type mouse (C57BL/6) testicular sections were stained with recombinant EGFP-MBD-NLS probe and counterstained with Hoechst 33342. Magnified images are shown on the bottom. Note that somatic cells (Leydig cells, yellow arrows) had higher levels of methylated DNA and heterochromatin compared to germ cells inside seminiferous tubules (nuclei on right-hand side of dotted line). The higher background of recombinant EGFP-MBD-NLS probe staining compared with that for the Methyl*RO* mouse is probably caused by nonspecific binding of the probe and/or the use of unfixed sections. EGFP-MBD-NLS probe signal is colored in pseudocolor (red).

See also Figure S1.

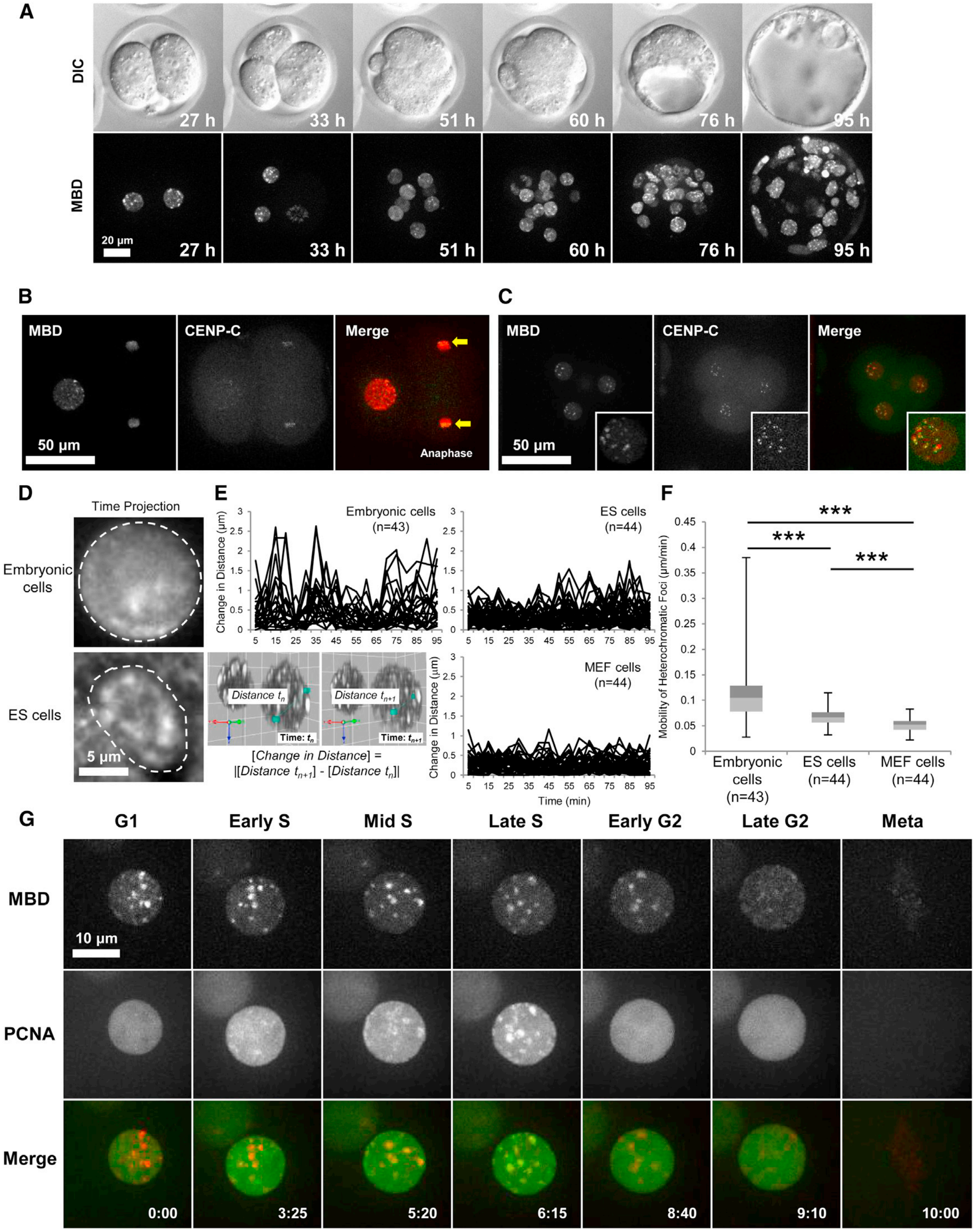


**Figure 5. Dynamic Changes in Patterns of Methylated DNA in Preimplantation Embryonic Nuclei**

(A) The time-lapse imaging of a Methyl*RO* embryo was performed using a confocal microscope equipped with a 30× silicone oil-immersion objective lens. Images were taken every hour from the zygote (0 hr) to blastocyst (119 hr) stage. MBD, mCherry-MBD-NLS.

(B) The mCherry-MBD-NLS probe marked pericentromeric heterochromatin. Two- to four-cell-stage division of Methyl*RO* embryo was imaged together with EGFP-CENP-C protein to label the centromere. Arrows indicate the centromeric regions of anaphase chromosomes.

(C) Interphase of four-cell-stage embryo was imaged together with CENP-C. Centromeres and mCherry-MBD-NLS foci located close together in interphase nuclei.

*(legend continued on next page)*

foci of trophectodermal cells became stronger in a time-dependent manner, together with the increase in nuclear size (Figures 6B and 6D–6G; Movie S8); moreover, there was no cell division, which probably reflects the endoreplication of these cells. These observations suggest that not only the DNA methylation status itself but also heterochromatin dynamics and stability are markers of the cellular differentiation status. Thus, we have demonstrated that Methyl*RO* can capture DNA methylation dynamics during differentiation in living cells and in mice.

## DISCUSSION

Accumulating evidence indicates that DNA methylation changes dynamically during development, differentiation, pathological processes, and in response to environmental cues (Bergman and Cedar, 2013; Feil and Fraga, 2011; Reik, 2007; Waterland and Jirtle, 2004). Hence, there is a strong need for new experimental approaches to study DNA methylation dynamics sequentially and quantitatively at the organismal level. In this report, we have shown clearly that the Methyl*RO* mouse is viable and fertile and can be used to visualize DNA methylation patterns without any fixation or treatments.

Consistent with previous reports by Kobayakawa et al. (2007), Tsumura et al. (2006), and Yamazaki et al. (2007), we have provided multiple evidences that the majority of the mCherry-MBD-NLS probe was concentrated at heterochromatic regions, especially at pericentromeric heterochromatin in preimplantation embryonic cells, ESCs, and somatic cells. Intriguingly, we have discovered that the heterochromatin of preimplantation embryos is highly dynamic because it did not stay at one certain position; rather, it changed its location dramatically in interphase nuclei. In addition, heterochromatic foci not only moved around inside nuclei but also disappeared depending on the cell-cycle status; in contrast, these foci never disappeared in ESCs, indicating that heterochromatin is already fixed, and this fixation actually occurred during the ESC-derivation process. Thus, heterochromatin fixation could be the reason for the difficulty in reversing ESCs to totipotent embryonic cell state (Beddington and Robertson, 1989; Morgani et al., 2013). Moreover, we succeeded in capturing the dynamic changes in DNA methylation status and its pattern in each cell lineage during the ESC-derivation process. Accordingly, these data suggest that the heterochromatin dynamics and stability can be markers of the cellular differentiation status, and further support the idea that chromatin plasticity decreases upon differentiation (Meshorer et al., 2006). Importantly, although we found that H2B signals can be used to calculate the heterochromatin indexes, the heterochromatin pattern was not so clear in visual as compared to MBD probe signals (Figures S3G and S3H). Therefore, it is not suitable to normalize MBD probe signal against that of H2B because this will cancel out the differences seen in the MBD (Figure S3I). Although the MBD reporter was designed to report DNA methylation, this result (the correlation with H2B-EGFP) suggests that the difference in MBD signals for some cell types may reflect a change in heterochromatin organization, and not necessarily in DNA methylation. On the other hand, this also calls for attention toward current conventional studies using immunohistochemistry because fixed cells do not provide information regarding the cell-cycle status or the differentiation process in single cells. Hence, Methyl*RO* mice will be useful for the study of the nuclear dynamics of heterochromatin during proliferation, differentiation, and development.

Using bisulfite sequencing and Southern blotting analyses, we demonstrated previously that the major and minor satellites of pericentromeric heterochromatin are hypomethylated only in germ cell lineages, whereas all somatic cells are hypermethylated, and that this DNA methylation profile is established during primordial germ cell development (Yamagata et al., 2007). This finding was further supported by the analysis of Methyl*RO* mouse sections because most somatic cells exhibited the typical puncta pattern of mCherry-MBD-NLS, whereas testicular germ cells had obviously weaker or no signals. Although many studies have documented extensively the active DNA demethylation during preimplantation development (Reik, 2007; Wu and Zhang, 2010), the pericentromeric

(D) Heterochromatin of embryonic cells (four-cell stage) was highly mobile. A total of 20 cellular nuclear images of 5 min intervals were projected at time-axis direction. Dotted lines indicate the boundary of the nucleus.
(E) Heterochromatin foci movement was more dynamic in embryonic cells. Distance of any two foci was measured, and time-dependent changes of distance were plotted as a line graph. The calculation formula to derive change in distance of randomly picked two foci is indicated in bottom-left image.
(F) Heterochromatic foci move faster in embryonic cells. A box-and-whisker plot of mobility of heterochromatic foci in different cell types is shown.
(G) Heterochromatin foci disappeared in G2 phase of preimplantation embryos. A Methyl*RO* embryo (four-cell stage) was imaged together with the EGFP-PCNA probe to assess its correlation with the cell cycle.
See also Figure S2 and Movies S1, S2, S3, S4, and S5.

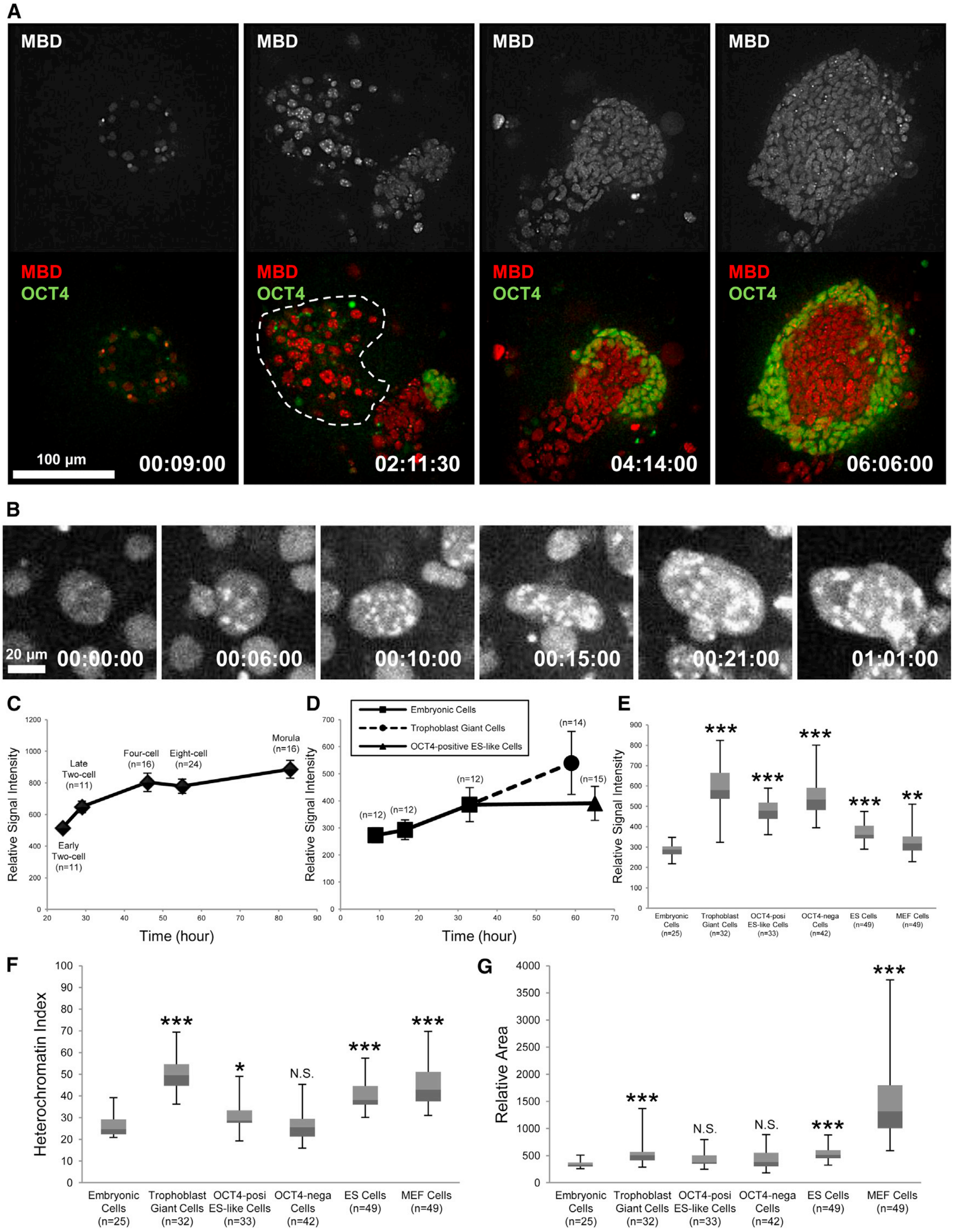


**Figure 6. DNA Methylation and Nuclear Organization Underwent Dynamic Changes during the ESC-Derivation Process**

(A) Time-lapse imaging of the ESC-derivation process using Methyl*RO* and OCT4-EGFP double-reporter embryos. OCT4-EGFP marked the pluripotent epiblast cell lineages that eventually become ESCs. The time after starting the observation is indicated at the bottom right (day:hour:minute). Nuclei surrounded by the dotted line indicate trophoblast giant cells.

(B) Dynamic nuclear remodeling of trophoblast giant cells. The time after starting the observation is indicated at the bottom right (day:hour:minute).

*(legend continued on next page)*

regions, which constitute 3.5% of the whole mouse genome that contains hypermethylated repeat sequences (Lehnertz et al., 2003; Waterston et al., 2002), are already hypomethylated, which seems to be the key epigenetic feature that distinguishes germ cells from somatic cells.

In addition to the use of the Methyl*RO* model as a reporter mouse for live-cell imaging, we have extended the application of this bioresource to MeDIP analysis. Conventional 5mC-mediated MeDIP is performed in vitro by binding extracted DNA with an anti-5mC antibody, which may yield unwanted results that do not reflect the in vivo status. In contrast, because the mCherry-MBD-NLS probe is expressed endogenously, we believe that it can capture the "exact moment" of DNA methylation dynamics. The use of an anti-RFP antibody for MeDIP-seq, and the fact that this reporter mouse is conditional, will allow us to perform cell-type- or tissue-specific MeDIP analysis by crossing with tissue-specific FLP Tg mice. Theoretically, this will result in an extremely low background, which is not possible with the conventional anti-5mC antibody-based methods.

In conclusion, we have provided multiple evidences to show that Methyl*RO* mice can capture the dynamic changes of the DNA methylation status both in vitro and in vivo. In particular, Methyl*RO* mice can be used not only in live-cell imaging analyses but also in RFP-mediated MeDIP-seq and cross-section observation analyses, which extend the applications of this bioresource. Hence, we believe that this mouse model will become a powerful tool as well as technique to study DNA methylation dynamics during development, differentiation, and in pathological processes that lead to diseases.

## EXPERIMENTAL PROCEDURES

### Quantification of Imaging Data

Imaging data were analyzed using the MetaMorph (version 7.7.2.0; Molecular Devices) and Volocity (version 6.2.1; PerkinElmer) software. Briefly, heterochromatin foci were randomly picked up and tracked manually, and distances between any two points in all combinations were measured three dimensionally using Volocity software. Heterochromatin movement was derived by subtracting distances using the following formula: change in distance = (distance $t_{n+1}$) − (distance $t_n$). Mobility (μm/min) was calculated by dividing change in distance by time (5 min). Average signal intensities, intensity SDs, and cell nucleus areas were calculated with MetaMorph software by drawing the region of interest around the nucleus manually based on 2D images projected in the z axis direction. The heterochromatin index was defined as a coefficient of variation of signal patterns and calculated using the following formula: heterochromatin index = SD of signal intensity / average signal intensity × 100. Statistical significance of each pair was calculated with the Steel-Dwass test, and statistical significance against embryonic cells was calculated with the Steel test, using the JMP software (version 10.0.2; SAS Institute). p values <0.05 were considered statistically significant and are indicated as follows: $^*p < 0.05$, $^{**}p < 0.01$, and $^{***}p < 0.001$. N.S. indicates "not significant." Cell culture, MeDIP analysis, generation of knockin mice, antibodies, histology, generation of recombinant EGFP-MBD-NLS probe, and live-cell imaging are provided in the Supplemental Experimental Procedures.

All animal experiments were approved by the Animal Care and Use Committee of the Research Institute for Microbial Diseases, Osaka University, Japan.

## SUPPLEMENTAL INFORMATION

Supplemental Information includes Supplemental Experimental Procedures, three figures, one table, and eight movies and can be found with this article online at http://dx.doi.org/10.1016/j.stemcr.2014.05.008.

## ACKNOWLEDGMENTS

We would like to thank Drs. Frank Costantini and Junji Takeda for providing the pBigT and *ROSA26*-targeting vector plasmids, Drs. Jan Ellenberg, Tomoya Kitajima, and Miho Ohsugi for the EGFP-CENP-C expression plasmid, Dr. Cristina M. Cardoso for the EGFP-PCNA expression plasmid, and Dr. Hitoshi Niwa for the *Oct4-EGFP* knockin-targeting vector. We would also like to thank Ms. Masumi Fujikawa for the Methyl*RO* mouse illustration and Mr. Masanaga Muto for help with cryosectioning. Finally, we express our greatest gratitude to Drs. Masaru Okabe and Masahito Ikawa for their encouragement, guidance, and support throughout this work. This study was supported in part by a Grant-in-Aid from the Ministry of Education, Culture, Sports, Science, and Technology of Japan.

Received: December 19, 2013
Revised: May 8, 2014
Accepted: May 10, 2014
Published: June 3, 2014

(C) Quantification of mCherry-MBD-NLS signal intensities during the preimplantation development. mCherry-MBD-NLS signals started to become visible from the early two-cell stage, reached their upper limit around the morula stage, and did not decrease during this period.
(D) Quantification of mCherry-MBD-NLS signal intensities during ESC-derivation processes. When an embryo was placed in ESC-derivation medium, signals increased in a time-dependent manner.
(E) Quantification of mCherry-MBD-NLS signal intensities in different cell types. Box-and-whisker plot of average signal intensity is shown.
(F) Quantification of the heterochromatin index in different cell types. Data are shown in box-and-whisker plot.
(G) Quantification of nuclear area in different cell types. Data are shown in box-and-whisker plot.
See also Figure S3 and Movies S6, S7, and S8.

## REFERENCES

Abe, T., Kiyonari, H., Shioi, G., Inoue, K., Nakao, K., Aizawa, S., and Fujimori, T. (2011). Establishment of conditional reporter mouse lines at ROSA26 locus for live cell imaging. Genesis *49*, 579–590.

Aguirre-Lavin, T., Adenot, P., Bonnet-Garnier, A., Lehmann, G., Fleurot, R., Boulesteix, C., Debey, P., and Beaujean, N. (2012). 3D-FISH analysis of embryonic nuclei in mouse highlights several abrupt changes of nuclear organization during preimplantation development. BMC Dev. Biol. *12*, 30.

Anders, S. (2009). Visualization of genomic data with the Hilbert curve. Bioinformatics *25*, 1231–1235.

Beddington, R.S., and Robertson, E.J. (1989). An assessment of the developmental potential of embryonic stem cells in the midgestation mouse embryo. Development *105*, 733–737.

Bergman, Y., and Cedar, H. (2013). DNA methylation dynamics in health and disease. Nat. Struct. Mol. Biol. *20*, 274–281.

Bird, A. (2002). DNA methylation patterns and epigenetic memory. Genes Dev. *16*, 6–21.

Clark, C., Palta, P., Joyce, C.J., Scott, C., Grundberg, E., Deloukas, P., Palotie, A., and Coffey, A.J. (2012). A comparison of the whole genome approach of MeDIP-seq to the targeted approach of the Infinium HumanMethylation450 BeadChip(®) for methylome profiling. PLoS One *7*, e50233.

Clouaire, T., and Stancheva, I. (2008). Methyl-CpG binding proteins: specialized transcriptional repressors or structural components of chromatin? Cell. Mol. Life Sci. *65*, 1509–1522.

Ehrlich, M. (2002). DNA methylation in cancer: too much, but also too little. Oncogene *21*, 5400–5413.

Ehrlich, M. (2009). DNA hypomethylation in cancer cells. Epigenomics *1*, 239–259.

Evans, M.J., and Kaufman, M.H. (1981). Establishment in culture of pluripotential cells from mouse embryos. Nature *292*, 154–156.

Feil, R., and Fraga, M.F. (2011). Epigenetics and the environment: emerging patterns and implications. Nat. Rev. Genet. *13*, 97–109.

Filipponi, D., Muller, J., Emelyanov, A., and Bulavin, D.V. (2013). Wip1 controls global heterochromatin silencing via ATM/BRCA1-dependent DNA methylation. Cancer Cell *24*, 528–541.

Frommer, M., McDonald, L.E., Millar, D.S., Collis, C.M., Watt, F., Grigg, G.W., Molloy, P.L., and Paul, C.L. (1992). A genomic sequencing protocol that yields a positive display of 5-methylcytosine residues in individual DNA strands. Proc. Natl. Acad. Sci. USA *89*, 1827–1831.

Fujihara, Y., Kaseda, K., Inoue, N., Ikawa, M., and Okabe, M. (2013). Production of mouse pups from germline transmission-failed knockout chimeras. Transgenic Res. *22*, 195–200.

Guenatri, M., Bailly, D., Maison, C., and Almouzni, G. (2004). Mouse centric and pericentric satellite repeats form distinct functional heterochromatin. J. Cell Biol. *166*, 493–505.

Guo, J.U., Su, Y., Zhong, C., Ming, G.L., and Song, H. (2011). Emerging roles of TET proteins and 5-hydroxymethylcytosines in active DNA demethylation and beyond. Cell Cycle *10*, 2662–2668.

Habibi, E., Brinkman, A.B., Arand, J., Kroeze, L.I., Kerstens, H.H., Matarese, F., Lepikhov, K., Gut, M., Brun-Heath, I., Hubner, N.C., et al. (2013). Whole-genome bisulfite sequencing of two distinct interconvertible DNA methylomes of mouse embryonic stem cells. Cell Stem Cell *13*, 360–369.

Ito, S., D'Alessio, A.C., Taranova, O.V., Hong, K., Sowers, L.C., and Zhang, Y. (2010). Role of Tet proteins in 5mC to 5hmC conversion, ES-cell self-renewal and inner cell mass specification. Nature *466*, 1129–1133.

Jørgensen, H.F., Adie, K., Chaubert, P., and Bird, A.P. (2006). Engineering a high-affinity methyl-CpG-binding protein. Nucleic Acids Res. *34*, e96.

Karmodiya, K., Krebs, A.R., Oulad-Abdelghani, M., Kimura, H., and Tora, L. (2012). H3K9 and H3K14 acetylation co-occur at many gene regulatory elements, while H3K14ac marks a subset of inactive inducible promoters in mouse embryonic stem cells. BMC Genomics *13*, 424.

Kobayakawa, S., Miike, K., Nakao, M., and Abe, K. (2007). Dynamic changes in the epigenomic state and nuclear organization of differentiating mouse embryonic stem cells. Genes Cells *12*, 447–460.

Kobayashi, H., Sakurai, T., Imai, M., Takahashi, N., Fukuda, A., Yayoi, O., Sato, S., Nakabayashi, K., Hata, K., Sotomaru, Y., et al. (2012). Contribution of intragenic DNA methylation in mouse gametic DNA methylomes to establish oocyte-specific heritable marks. PLoS Genet. *8*, e1002440.

Lehnertz, B., Ueda, Y., Derijck, A.A., Braunschweig, U., Perez-Burgos, L., Kubicek, S., Chen, T., Li, E., Jenuwein, T., and Peters, A.H. (2003). Suv39h-mediated histone H3 lysine 9 methylation directs DNA methylation to major satellite repeats at pericentric heterochromatin. Curr. Biol. *13*, 1192–1200.

Leonhardt, H., Rahn, H.P., Weinzierl, P., Sporbert, A., Cremer, T., Zink, D., and Cardoso, M.C. (2000). Dynamics of DNA replication factories in living cells. J. Cell Biol. *149*, 271–280.

Li, E., Bestor, T.H., and Jaenisch, R. (1992). Targeted mutation of the DNA methyltransferase gene results in embryonic lethality. Cell *69*, 915–926.

Meshorer, E., Yellajoshula, D., George, E., Scambler, P.J., Brown, D.T., and Misteli, T. (2006). Hyperdynamic plasticity of chromatin proteins in pluripotent embryonic stem cells. Dev. Cell *10*, 105–116.

Mikkelsen, T.S., Wakefield, M.J., Aken, B., Amemiya, C.T., Chang, J.L., Duke, S., Garber, M., Gentles, A.J., Goodstadt, L., Heger, A., et al.; Broad Institute Genome Sequencing Platform; Broad Institute Whole Genome Assembly Team (2007). Genome of the marsupial *Monodelphis domestica* reveals innovation in non-coding sequences. Nature *447*, 167–177.

Miné-Hattab, J., and Rothstein, R. (2012). Increased chromosome mobility facilitates homology search during recombination. Nat. Cell Biol. *14*, 510–517.

Morgani, S.M., Canham, M.A., Nichols, J., Sharov, A.A., Migueles, R.P., Ko, M.S., and Brickman, J.M. (2013). Totipotent embryonic stem cells arise in ground-state culture conditions. Cell Reports *3*, 1945–1957.

Nichols, J., and Smith, A. (2011). The origin and identity of embryonic stem cells. Development *138*, 3–8.

Ohki, I., Shimotake, N., Fujita, N., Jee, J., Ikegami, T., Nakao, M., and Shirakawa, M. (2001). Solution structure of the methyl-CpG

binding domain of human MBD1 in complex with methylated DNA. Cell *105*, 487–497.

Okabe, M., Ikawa, M., Kominami, K., Nakanishi, T., and Nishimune, Y. (1997). 'Green mice' as a source of ubiquitous green cells. FEBS Lett. *407*, 313–319.

Okano, M., Bell, D.W., Haber, D.A., and Li, E. (1999). DNA methyltransferases Dnmt3a and Dnmt3b are essential for de novo methylation and mammalian development. Cell *99*, 247–257.

Reik, W. (2007). Stability and flexibility of epigenetic gene regulation in mammalian development. Nature *447*, 425–432.

Saitoh, H., Tomkiel, J., Cooke, C.A., Ratrie, H., 3rd, Maurer, M., Rothfield, N.F., and Earnshaw, W.C. (1992). CENP-C, an autoantigen in scleroderma, is a component of the human inner kinetochore plate. Cell *70*, 115–125.

Sasaki, H., and Matsui, Y. (2008). Epigenetic events in mammalian germ-cell development: reprogramming and beyond. Nat. Rev. Genet. *9*, 129–140.

Seki, Y., Hayashi, K., Itoh, K., Mizugaki, M., Saitou, M., and Matsui, Y. (2005). Extensive and orderly reprogramming of genome-wide chromatin modifications associated with specification and early development of germ cells in mice. Dev. Biol. *278*, 440–458.

Shcherbo, D., Merzlyak, E.M., Chepurnykh, T.V., Fradkov, A.F., Ermakova, G.V., Solovieva, E.A., Lukyanov, K.A., Bogdanova, E.A., Zaraisky, A.G., Lukyanov, S., and Chudakov, D.M. (2007). Bright far-red fluorescent protein for whole-body imaging. Nat. Methods *4*, 741–746.

Shioi, G., Kiyonari, H., Abe, T., Nakao, K., Fujimori, T., Jang, C.W., Huang, C.C., Akiyama, H., Behringer, R.R., and Aizawa, S. (2011). A mouse reporter line to conditionally mark nuclei and cell membranes for in vivo live-imaging. Genesis *49*, 570–578.

Smith, Z.D., Chan, M.M., Mikkelsen, T.S., Gu, H., Gnirke, A., Regev, A., and Meissner, A. (2012). A unique regulatory phase of DNA methylation in the early mammalian embryo. Nature *484*, 339–344.

Soriano, P. (1999). Generalized lacZ expression with the ROSA26 Cre reporter strain. Nat. Genet. *21*, 70–71.

Srinivas, S., Watanabe, T., Lin, C.S., William, C.M., Tanabe, Y., Jessell, T.M., and Costantini, F. (2001). Cre reporter strains produced by targeted insertion of EYFP and ECFP into the ROSA26 locus. BMC Dev. Biol. *1*, 4.

Toyooka, Y., Shimosato, D., Murakami, K., Takahashi, K., and Niwa, H. (2008). Identification and characterization of subpopulations in undifferentiated ES cell culture. Development *135*, 909–918.

Tsumura, A., Hayakawa, T., Kumaki, Y., Takebayashi, S., Sakaue, M., Matsuoka, C., Shimotohno, K., Ishikawa, F., Li, E., Ueda, H.R., et al. (2006). Maintenance of self-renewal ability of mouse embryonic stem cells in the absence of DNA methyltransferases Dnmt1, Dnmt3a and Dnmt3b. Genes Cells *11*, 805–814.

Waterland, R.A., and Jirtle, R.L. (2003). Transposable elements: targets for early nutritional effects on epigenetic gene regulation. Mol. Cell. Biol. *23*, 5293–5300.

Waterland, R.A., and Jirtle, R.L. (2004). Early nutrition, epigenetic changes at transposons and imprinted genes, and enhanced susceptibility to adult chronic diseases. Nutrition *20*, 63–68.

Waterston, R.H., Lindblad-Toh, K., Birney, E., Rogers, J., Abril, J.F., Agarwal, P., Agarwala, R., Ainscough, R., Alexandersson, M., An, P., et al.; Mouse Genome Sequencing Consortium (2002). Initial sequencing and comparative analysis of the mouse genome. Nature *420*, 520–562.

Wu, S.C., and Zhang, Y. (2010). Active DNA demethylation: many roads lead to Rome. Nat. Rev. Mol. Cell Biol. *11*, 607–620.

Yamagata, K., Yamazaki, T., Miki, H., Ogonuki, N., Inoue, K., Ogura, A., and Baba, T. (2007). Centromeric DNA hypomethylation as an epigenetic signature discriminates between germ and somatic cell lineages. Dev. Biol. *312*, 419–426.

Yamagata, K., Ueda, J., Mizutani, E., Saitou, M., and Wakayama, T. (2010). Survival and death of epiblast cells during embryonic stem cell derivation revealed by long-term live-cell imaging with an Oct4 reporter system. Dev. Biol. *346*, 90–101.

Yamaji, M., Ueda, J., Hayashi, K., Ohta, H., Yabuta, Y., Kurimoto, K., Nakato, R., Yamada, Y., Shirahige, K., and Saitou, M. (2013). PRDM14 ensures naive pluripotency through dual regulation of signaling and epigenetic pathways in mouse embryonic stem cells. Cell Stem Cell *12*, 368–382.

Yamazaki, T., Yamagata, K., and Baba, T. (2007). Time-lapse and retrospective analysis of DNA methylation in mouse preimplantation embryos by live cell imaging. Dev. Biol. *304*, 409–419.

Yoder, J.A., Walsh, C.P., and Bestor, T.H. (1997). Cytosine methylation and the ecology of intragenomic parasites. Trends Genet. *13*, 335–340.

**Stem Cell Reports, Volume *2***

**Supplemental Information**

# Heterochromatin Dynamics during the Differentiation Process Revealed by the DNA Methylation Reporter Mouse, Methyl*RO*

**Jun Ueda, Kazumitsu Maehara, Daisuke Mashiko, Takako Ichinose, Tatsuma Yao, Mayuko Hori, Yuko Sato, Hiroshi Kimura, Yasuyuki Ohkawa, and Kazuo Yamagata**

**Supplemental Figure Legends**

Figure S1, related to Figure 4. Generation of the ROSA26-H2B-mCherry reporter mouse. (A) Targeting of H2B-mCherry to the *ROSA26* locus. Top to bottom: pBigT, a plasmid containing an FRT-flanked cassette with a promoter-less Neo selectable marker and a tpA transcriptional stop sequence, into which the H2B-mCherry was cloned; pROSA26PA, containing genomic *ROSA26* sequences for homologous recombination, and a diphtheria toxin gene (PGK-DTA) for negative selection in ES cells; the wild-type *ROSA26* locus, with the location of the primers indicated; structure of the targeted locus; and structure of the targeted locus after Flipase (FLP)-mediated excision of the FRT-flanked (Neo, tpA) cassette. The FRT sites are indicated by solid arrowheads. (B) Schematic representation of H2B-mCherry. (C) Validation of genotypes of ES cells by PCR. The insertion of short and long arms was confirmed by PCR using the primers indicated in (A). (D) Genotyping of the ROSA26-H2B-mCherry mouse by PCR. WT, wild-type; Het, heterozygous knock-in; Homo, homozygous knock-in. (E) The H2B-mCherry protein was expressed only when the neomycin resistance gene cassette was removed by FLP. The presence of H2B-mCherry was confirmed by immunoblotting using an anti-RFP antibody in ES cells. An anti-OCT4 antibody was used as a loading control. (F) ROSA26-H2B-mCherry was expressed ubiquitously in adult mouse organs. The panels show the expression of ROSA26-H2B-mCherry and CAG-EGFP genes in various organs, including the brain, heart, lung, kidney, pancreas, testis, spleen, liver, thymus, and seminal vesicles. Both control and ROSA26-H2B-mCherry mice were positive for EGFP because these mice were generated using green ES cells (Fujihara et al., 2013). Although CAG is considered to be ubiquitous, there was no obvious EGFP signal in liver.

Figure S2, related to Figure 5. (A) Three-dimensional images of a blastocyst-stage Methyl*RO* embryo. The arrow indicates a cell with no obvious mCherry-MBD-NLS foci. A confocal microscope equipped with a 60× silicone oil-immersion objective lens was used to obtain the z-axis images, and three-dimensional images were generated computationally using the MetaMorph software. Numbers indicate angles. (B) Time-lapse imaging of Methyl*RO* ES cells. Whole chromosomes were labeled with H2B-EGFP expressed from the *ROSA26* locus. Note that the mCherry-MBD-NLS probe was highly enriched in pericentromeric regions (arrows). Furthermore, mCherry-MBD-NLS foci remained visible throughout the cell cycle. This was in sharp contrast with the preimplantation embryonic development described in Figure 4, in which mCherry-MBD-NLS foci disappeared in G2 phase of cell cycle. ES cells were cultured in LIF and FCS-containing medium, and photos were taken every 10 min using a confocal microscope equipped with a 60× silicone oil-immersion objective lens as described in Supplemental Experimental Procedures. The time after starting observation is indicated at the bottom right of the panel (hour:minute).

Figure S3, related to Figure 6. Generation of *Oct4-EGFP* knock-in mice. (A) Targeting of OCT4-EGFP to the *Oct4* gene locus. Targeting vector was kindly gift of Dr. Hitoshi Niwa (RIKEN CDB) (Toyooka et al., 2008). (B) Validation of genotypes by PCR in ES cells. The insertion of short and long arms was confirmed by PCR using the primers indicated in (A). Knock-in mouse was generated using these ES cells. (C) Genotyping of *Oct4-EGFP* knock-in mice by PCR. (D) OCT4-EGFP protein expression was confirmed by western blotting using ES cell whole protein extracts. WT, wild-type; Het, heterozygous knock-in. (E) Time-lapse images of an *Oct4-EGFP* knock-in embryo (upper embryo). Bottom embryo is negative for *Oct4-EGFP*. The time after starting observation is indicated at the bottom of the panel (hour). OCT4-EGFP signal becomes visible from four-cell stage embryo (Supplemental Movie 6). (F) Confocal image of *Oct4-EGFP* knock-in ES cells. (G) Confocal images of H2B-EGFP- and mCherry-MBD-NLS-expressing ES cells and differentiated cells. Differentiation was induced by culturing ES cells with retinoic acid containing medium for 7 days. (H) Heterochromatin index can be induced using H2B-EGFP probe. Heterochromatin indexes of ES and differentiated cells were calculated using either mCherry-MBD-NLS or H2B-EGFP probes. Statistical significance was calculated using Mann-Whitney U test. (I) Normalization of mCherry-MBD-NLS with H2B-EGFP signals cancel out the difference between ES and differentiated cells. Statistical significance was calculated using Mann-Whitney U test.

**Supplemental Table**

Supplemental Table S1. Genotyping primers used in this study.

| | Primer Name | Sequence |
|---|---|---|
| 1 | ROSA26-Short Arm-Fw | 5′-CCT AAA GAA GAG GCT GTG CTT TGG-3′ |
| 2 | MBD-Rev | 5′-TTA CGG GGC CTC CTT CCT GAC CTC ACC ACT CT-3′ |
| 3 | MBD-Fw | 5′-AAG ACT CGG AAA CGT CAG GTT GGA CCC CAG-3′ |
| 4 | ROSA26-Long Arm-Rev | 5′-GTA GTT ACT CCA CTT TCA AGT TCC TTA TAA-3′ |
| 5 | ROSA26-NotI-Fw | 5′-GAG CGG CCG CCC ACC CTC CCC TTC CTC TGG-3′ |
| 6 | ROSA26-NruI-Rev | 5′-CCT CGC GAC ACT GTA TTT CAT ACT GTA GTA-3′ |
| 7 | H2B-Rev | 5′-TGC GCC TTA GTC ACC GCC TTC TTG GAG CCC-3′ |
| 8 | mCherry-Fw | 5′-TCC ACC GGC GGC ATG GAC GAG CTG TAC AAG-3′ |
| 9 | Oct4-Short Arm-Fw | 5′-GGG CGG GGC CAG AGG TCA AGG CTA GAG GGT-3′ |
| 10 | EGFP-Rev | 5′-TTA CTT GTA CAG CTC GTC CAT GCC G-3′ |
| 11 | EGFP-Fw | 5′-ATG GTG AGC AAG GGC GAG GAG CTG TT-3′ |
| 12 | Oct4-Rev | 5′-CCT CAG AGG CCA CTC AGC ACT CTC TCC TCT-3′ |
| 13 | Oct4-Ex5-Fw | 5′-GTG GTT CGA GTA TGG TTC TGT AAC CGG CGC-3′ |
| 14 | Oct4-Ex5-Rev | 5′-GCT ATC TAC TGT GTG TCC CAG TCT TTA TTT-3′ |

**Supplemental Movies**

Supplemental Movie S1, related to Figure 4. Time-lapse imaging of a Methyl*RO* embryo from the one-cell zygote to the expanded blastocyst stage. Zygotic activation of mCherry-MBD-NLS started from the two-cell stage.

Supplemental Movie S2, related to Figure 4. Three dimensional image of a blastocyst stage Methyl*RO* embryo.

Supplemental Movie S3, related to Figure 4. Time-lapse imaging of EGFP-CENP-C and mCherry-MBD-NLS in a Methyl*RO* embryo from the two-cell to the eight-cell stage.

Supplemental Movie S4, related to Figure 4. Time-lapse imaging of EGFP-PCNA and mCherry-MBD-NLS in a Methyl*RO* embryo from the two-cell to the eight-cell stage.

Supplemental Movie S5, related to Figure 4. Time-lapse imaging of H2B-EGFP- and mCherry-MBD-NLS-expressing ES cells.

Supplemental Movie S6, related to Figure 5. Time-lapse imaging of an *Oct4-EGFP* knock-in embryo from the one-cell zygote to the expanded blastocyst stage.

Supplemental Movie S7, related to Figure 5. Time-lapse imaging of the ES cell derivation process in an OCT4-EGFP and mCherry-MBD-NLS double-positive embryo.

Supplemental Movie S8, related to Figure 5. Time-lapse imaging of a trophectodermal cell differentiating into a trophoblast giant cell.

**Supplemental Experimental Procedures**

*Cell culture*

Mouse ES cells were grown on top of mouse embryonic fibroblasts and maintained in medium containing 20% fetal calf serum and 500 U/mL LIF. Cell culture was performed in 5% $CO_2$ at 37°C. ES cell derivation was performed as described previously (Yamagata et al., 2010). For differentiation of ES cells, cells were cultured in 10 μM all-trans retinoic acid (Nacalai Tesque Co., Ltd., Japan) and 10% fetal calf serum containing medium for 7 days.

*MeDIP analysis*

After fixation with 1% paraformaldehyde, cells were harvested with a cell scraper and samples were processed for MeDIP analysis. Briefly, the cross-linked chromatin was sheared by sonication (Bioruptor, COSMO BIO), and MNase treatment (final concentration 10 U/mL). The insoluble materials were removed by centrifugation at 15,000 rpm for 10 min and supernatants were diluted with ChIP buffer (10 mM Tris-HCl pH 8.0, 200 mM KCl, 1 mM $CaCl_2$, 0.5% NP40) containing protease inhibitors. Immunoprecipitations were performed overnight in 4°C using antibody-conjugated Dynabeads® (Life Technologies Inc., CA, USA), and mixtures were extensively washed with Wash buffer (10 mM Tris-HCl pH 8.0, 500 mM KCl, 1 mM $CaCl_2$, 0.5% NP40). The bound chromatin fragments were released from the beads by adding ChIP Elution buffer (50 mM Tris-HCl pH8.0, 10 mM EDTA, 1% SDS) and incubated in 50°C for 1 hour in the presence of 2% proteinase K. MeDIPed DNA was purified by PCR purification kit (Qiagen N.V., Netherlands) and sequenced by the HiSeq 1000 (Illumina, Inc., CA, USA). The reads (Input 57,423,576 reads; 5mC 58,942,039 reads; RFP 61,096,178 reads) were aligned to the mouse genome (mm9) using the bowtie (version 0.12.7) software (parameter: -v 3 -m 1) and only uniquely mapped reads were used for calculation (percentages of mapped reads were 75.5% for Input, 66.7% for 5mC, and 65.9% for RFP, respectively). The normalized ChIP-seq signal was calculated by subtracting the Reads Per Million mapped reads (RPM) (Mortazavi et al., 2008) of the Input signal from the IPed signal. The signals were calculated at 1 kbp genomic intervals with a 10 kbp window. Hilbert curves of the signals were generated using the HilbertVis software, as described previously (Anders, 2009). MeDIP-seq data were deposited with the GEO accession code GSE57024.

*Generation of knock-in mice*

For the generation of Methyl*RO* and *ROSA26-H2B-mCherry* mice, targeting vectors were linearized by *KpnI* and electroporated into C57BL/6N ES cells (Fujihara et al., 2013). Genotypes were verified by PCR and ES cells with normal karyotype were injected into eight-cell-stage Slc:ICR strain embryos to generate chimeric mice. Mature male chimeric mice were first crossed

with C57BL/6N female mice to obtain F1 mice; subsequently, the neomycin cassette was excised from the *ROSA26* locus with FLP to induce the expression of either mCherry-MBD-NLS or H2B-mCherry in the whole body. The following primers were used to genotype Methyl*RO* ES cells: 'ROSA26-Short Arm-Fw' and 'MBD-Rev' were used to detect the insertion of the short arm, and 'MBD-Fw' and 'ROSA26-Long Arm-Rev' were used to detect the insertion of the long arm. The following three primers were used to genotype the Methyl*RO* mouse: 'ROSA26-NotI-Fw', 'ROSA26-NruI-Rev', and 'MBD-Rev'. To genotype *ROSA26-H2B-mCherry* ES cells, the following primers were used: 'ROSA26-Short Arm-Fw' and 'H2B-Rev' were used to detect the insertion of the short arm, and 'mCherry-Fw' and 'ROSA26-Long Arm-Rev' were used to detect the insertion of the long arm. The following three primers were used to genotype the *ROSA26-H2B-mCherry* mouse: 'ROSA26-NotI-Fw', 'ROSA26-NruI-Rev', and 'H2B-Rev'. For the generation of the *Oct4-EGFP* knock-in mouse, a targeting vector described previously was used (Toyooka et al., 2008). The following primers were used to genotype *Oct4-EGFP* ES cells: 'Oct4-Short Arm-Fw' and 'EGFP-Rev' were used to detect the insertion of the short arm, and 'EGFP-Fw' and 'Oct4-Rev' were used to detect the insertion of the long arm. The following three primers were used to genotype the *Oct4-EGFP* knock-in mouse: 'Oct4-Ex5-Fw', 'Oct4-Ex5-Rev', and 'EGFP-Rev'. Primer sequences are available in Supplemental Table S1. Methyl*RO*, conditional Methyl*RO*, *ROSA26-H2B-mCherry*, conditional *ROSA26-H2B-mCherry*, and *Oct4-EGFP* knock-in mice are available from the RIKEN BioResource Center. All animal experiments were approved by the Animal Care and Use Committee of the Research Institute for Microbial Diseases, Osaka University, Japan.

*Antibodies*

Anti-RFP (Cat. No. 632496, Takara Bio Inc., Japan), anti-GFP (Hayashi and Shirao, 1999), and anti-OCT4 (sc-5279, Santa Cruz Biotechnology, Inc., CA, USA) antibodies were used for western blotting. Anti-RFP (M165-3, MBL Co., Ltd., Japan) and anti-5mC (MAb-006-100, Diagenode Inc., USA) antibodies were used for MeDIP analysis.

*Histology*

E12.5 fetuses were obtained by crossing C57BL/6N females with Methyl*RO* heterozygous males and fixed with 4% paraformaldehyde at 4°C overnight. To prepare sections, fixed fetuses or organs were equilibrated in PBS (−) with 6.8% sucrose, dehydrated in acetone for 1 h, and embedded in glycol methacrylate (Technovit 8100; Heraeus Kulzer GmbH, Germany). Sections were cut at a thickness of 5 μm. For staining of testis section with recombinant EGFP-MBD-NLS probe, freshly prepared unfixed testes were embedded into O.C.T. compound (Sakura FineTek, Tokyo, Japan) and frozen. Then these samples were sectioned at a thickness of 8 μm, fixed with ethanol, blocked and stained with EGFP-MBD-NLS probe in 4.2 ng/mL concentration. All sections were stained with

Hoechst 33342 (Life Technologies Inc., CA, USA) and were imaged using an ECLIPSE Ti inverted confocal microscope (Nikon Co., Japan).

*Generation of recombinant EGFP-MBD-NLS probe*

EGFP-MBD-NLS was produced in bacteria and purified by column chromatography. EcoRI-NotI fragment containing EGFP-MBD from pcDNA3.1 GFP-MBD-NLS poly A (Yamagata et al., 2005) was cloned into pGEX-6P-1 (GE Healthcare) to express glutathione-S-transferase (GST) fusion protein. E. coli BL21 strain harboring the resulting plasmid was grown at 25°C and the expression of GST-EGFP-MBD-NLS was induced by addition of 0.1 mM isopropyl β-D-1-thiogalactopyranoside (7 h; 25°C). Cells were harvested by centrifugation, lysed in 50 mM Tris-HCl [pH 8.0], 150 mM NaCl, protease inhibitor cocktail (Nacalai Tesque), 0.2 mg/mL lysozyme (Seikagaku Kogyo), and 1% Triton X-100 by sonication. After centrifugation (8,900 ×g; 20 min; 4°C), the supernatant was mixed with Glutathione-Sepharose beads (GE Healthcare; 2 mL beads per 200 mL cell culture) overnight at 4°C. After washing beads with 0.1 M sodium phosphate [pH 7.0] buffer containing 1 M NaCl, GST-EGFP-MBD was eluted with 100 mM glutathione and digested with Prescision protease (31 units/mL; GE Healthcare) overnight at 4°C. GST moiety was absorbed by incubating with Glutathione-Sepharose beads. EGFP-MBD was further purified using a HiLoad Superdex75pg gel filtration column, concentrated up to 0.6 mg/mL in PBS, and stored at -80°C in small aliquots.

*Live-cell imaging*

Mouse oocyte preparation, *in vitro* fertilization (IVF), microinjection, and imaging were described previously (Yamagata et al., 2009; Yamagata and Ueda, 2013). Briefly, fertilized oocytes at the anaphase II-telophase II stage were injected with 100 µg/mL of EGFP-CENP-C (Kitajima et al., 2011) or 10 µg/mL of EGFP-PCNA (Leonhardt et al., 2000) probe mRNA, placed on the imaging system, and imaged at 37°C with 5% $CO_2$ in air conditions. A conventional inverted microscope (IX-71, Olympus Co., Japan) equipped with a Nipkow disk confocal unit (CSU-X1, Yokogawa Electric Co., Japan), electron-multiplying charge-coupled device camera (iXON3 DU897E-CS0-#BV-Y, Andor Technology plc., UK), silicon oil-immersion objective (30× or 60×) lenses (Olympus Co., Japan) was used for live-cell imaging. Live-cell imaging of the ES cell derivation process was performed as described previously (Yamagata et al., 2010).

**Supplemental References**

Anders, S. (2009). Visualization of genomic data with the Hilbert curve. Bioinformatics *25*, 1231-1235.

Fujihara, Y., Kaseda, K., Inoue, N., Ikawa, M., and Okabe, M. (2013). Production of mouse pups from germline transmission-failed knockout chimeras. Transgenic Res *22*, 195-200.

Hayashi, K., and Shirao, T. (1999). Change in the shape of dendritic spines caused by overexpression of drebrin in cultured cortical neurons. J Neurosci *19*, 3918-3925.

Kitajima, T.S., Ohsugi, M., and Ellenberg, J. (2011). Complete kinetochore tracking reveals error-prone homologous chromosome biorientation in mammalian oocytes. Cell *146*, 568-581.

Leonhardt, H., Rahn, H.P., Weinzierl, P., Sporbert, A., Cremer, T., Zink, D., and Cardoso, M.C. (2000). Dynamics of DNA replication factories in living cells. J Cell Biol *149*, 271-280.

Mortazavi, A., Williams, B.A., McCue, K., Schaeffer, L., and Wold, B. (2008). Mapping and quantifying mammalian transcriptomes by RNA-Seq. Nat Methods *5*, 621-628.

Toyooka, Y., Shimosato, D., Murakami, K., Takahashi, K., and Niwa, H. (2008). Identification and characterization of subpopulations in undifferentiated ES cell culture. Development *135*, 909-918.

Yamagata, K., Suetsugu, R., and Wakayama, T. (2009). Long-term, six-dimensional live-cell imaging for the mouse preimplantation embryo that does not affect full-term development. J Reprod Dev *55*, 343-350.

Yamagata, K., and Ueda, J. (2013). Long-term live-cell imaging of mammalian preimplantation development and derivation process of pluripotent stem cells from the embryos. Dev Growth Differ *55*, 378-389.

Yamagata, K., Ueda, J., Mizutani, E., Saitou, M., and Wakayama, T. (2010). Survival and death of epiblast cells during embryonic stem cell derivation revealed by long-term live-cell imaging with an Oct4 reporter system. Dev Biol *346*, 90-101.

Yamagata, K., Yamazaki, T., Yamashita, M., Hara, Y., Ogonuki, N., and Ogura, A. (2005). Noninvasive visualization of molecular events in the mammalian zygote. Genesis *43*, 71-79.

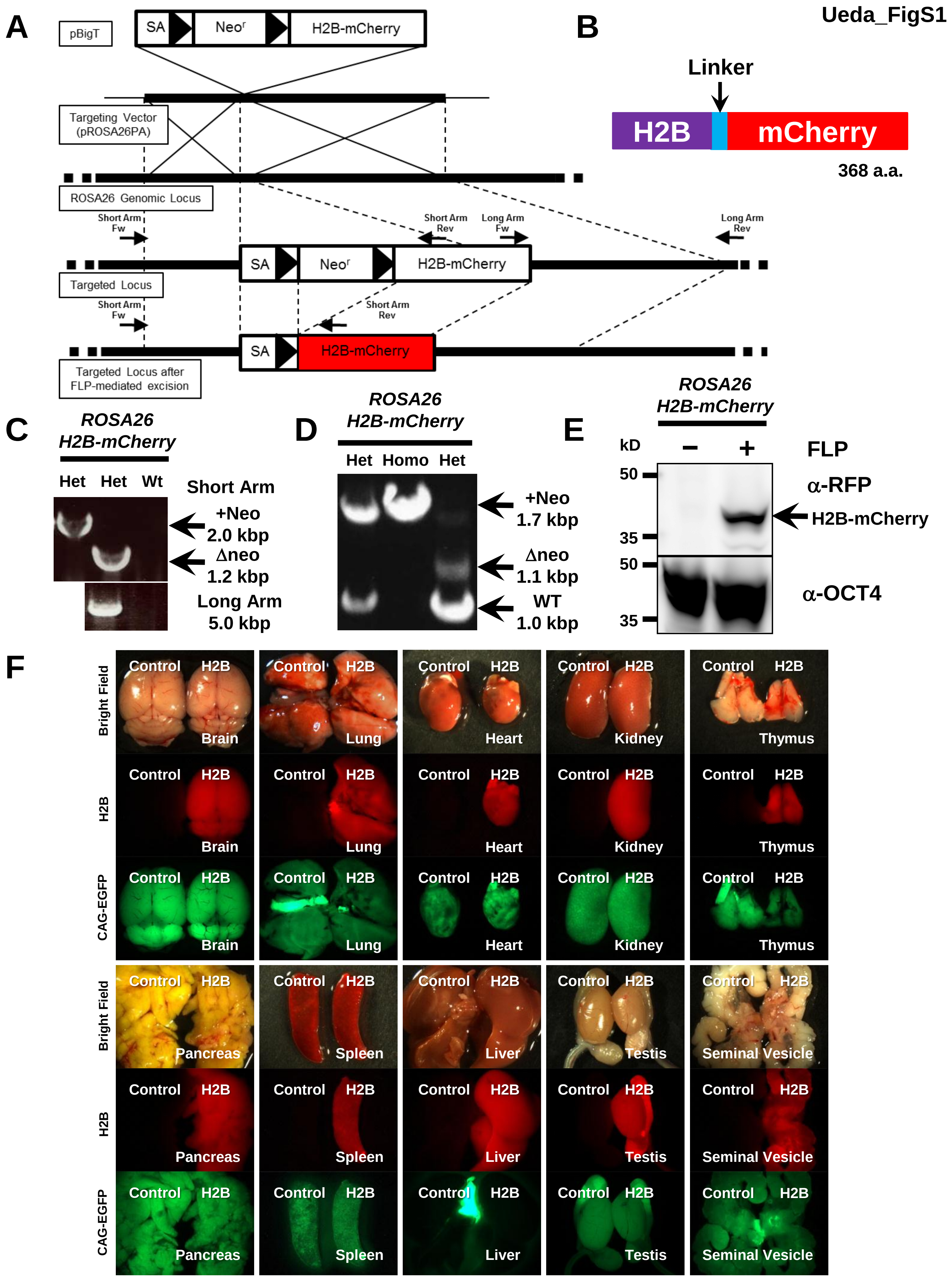
Ueda_FigS1
A
pBigT
SA
Neo^r
H2B-mCherry
Targeting Vector (pROSA26PA)
ROSA26 Genomic Locus
Short Arm Fw
Short Arm Rev
Long Arm Fw
Long Arm Rev
Targeted Locus
Targeted Locus after FLP-mediated excision
B
Linker
H2B
mCherry
368 a.a.
C
ROSA26 H2B-mCherry
Het Het Wt
Short Arm
+Neo 2.0 kbp
Δneo 1.2 kbp
Long Arm 5.0 kbp
D
ROSA26 H2B-mCherry
Het Homo Het
+Neo 1.7 kbp
Δneo 1.1 kbp
WT 1.0 kbp
E
ROSA26 H2B-mCherry
kD
− +
FLP
50
35
α-RFP
H2B-mCherry
α-OCT4
F
Bright Field
H2B
CAG-EGFP
Control H2B
Brain
Lung
Heart
Kidney
Thymus
Pancreas
Spleen
Liver
Testis
Seminal Vesicle

Ueda_FigS2

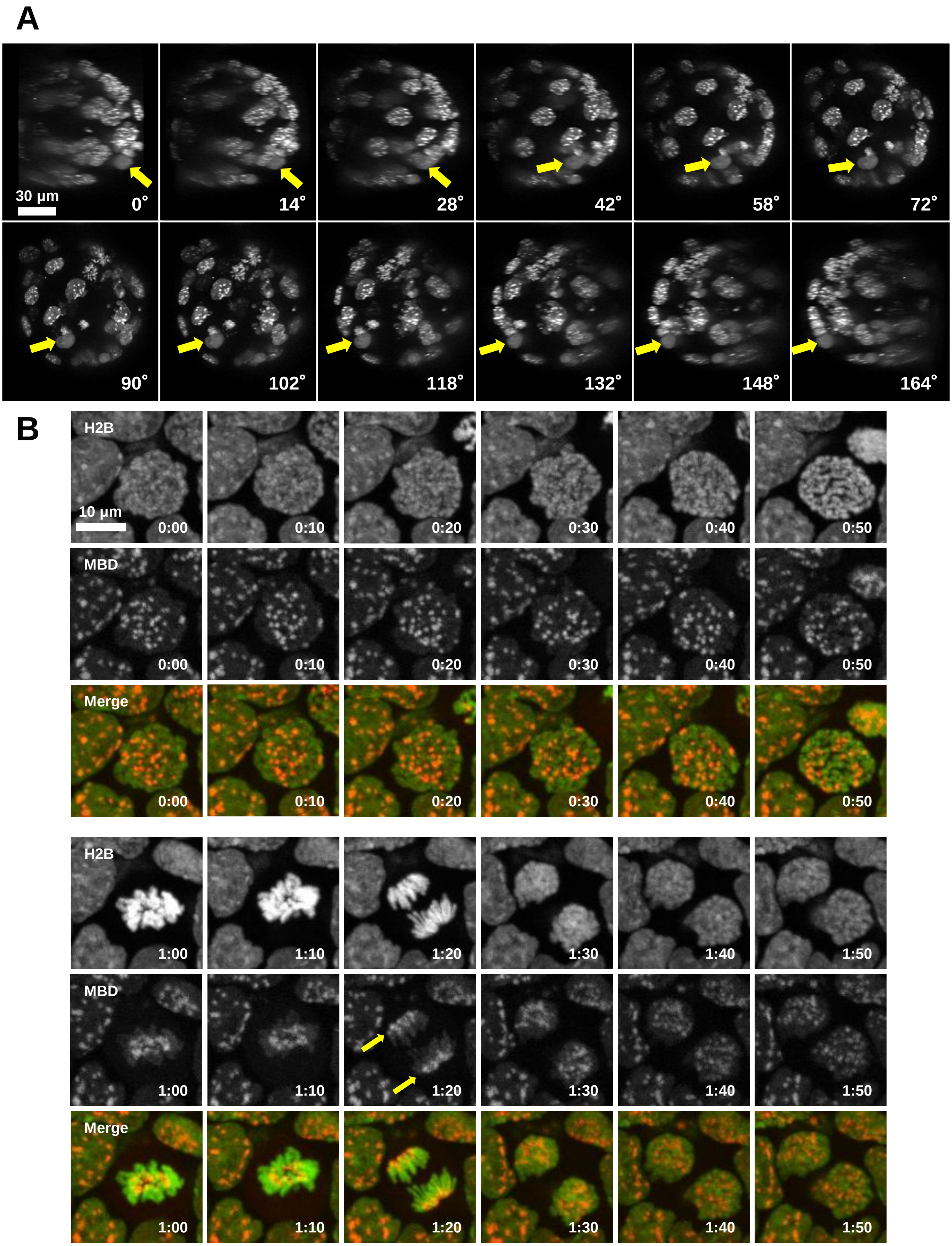
A
30 μm
0°
14°
28°
42°
58°
72°
90°
102°
118°
132°
148°
164°
B
H2B
MBD
Merge
10 μm
0:00
0:10
0:20
0:30
0:40
0:50
1:00
1:10
1:20
1:30
1:40
1:50

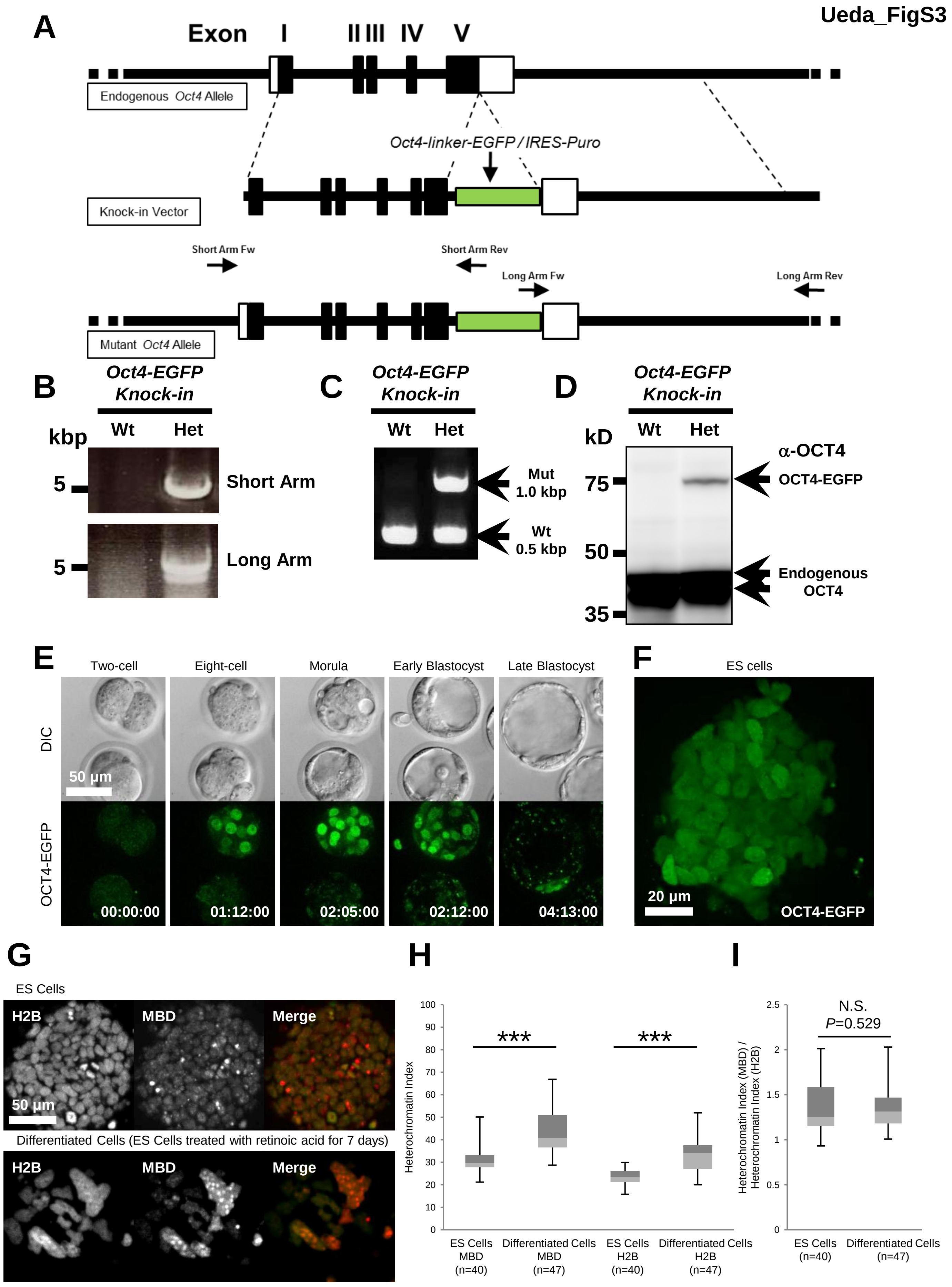
Ueda_FigS3
A
Exon I II III IV V
Endogenous Oct4 Allele
Oct4-linker-EGFP / IRES-Puro
Knock-in Vector
Short Arm Fw
Short Arm Rev
Long Arm Fw
Long Arm Rev
Mutant Oct4 Allele
B
Oct4-EGFP Knock-in
kbp
Wt
Het
5
Short Arm
5
Long Arm
C
Oct4-EGFP Knock-in
Wt
Het
Mut 1.0 kbp
Wt 0.5 kbp
D
Oct4-EGFP Knock-in
kD
Wt
Het
α-OCT4
75
OCT4-EGFP
50
Endogenous OCT4
35
E
Two-cell
Eight-cell
Morula
Early Blastocyst
Late Blastocyst
DIC
50 μm
OCT4-EGFP
00:00:00
01:12:00
02:05:00
02:12:00
04:13:00
F
ES cells
20 μm
OCT4-EGFP
G
ES Cells
H2B
MBD
Merge
50 μm
Differentiated Cells (ES Cells treated with retinoic acid for 7 days)
H2B
MBD
Merge
H
Heterochromatin Index
100
90
80
70
60
50
40
30
20
10
0
***
***
ES Cells MBD (n=40)
Differentiated Cells MBD (n=47)
ES Cells H2B (n=40)
Differentiated Cells H2B (n=47)
I
Heterochromatin Index (MBD) / Heterochromatin Index (H2B)
2.5
2
1.5
1
0.5
0
N.S.
P=0.529
ES Cells (n=40)
Differentiated Cells (n=47)
